# Social and Emotional Learning Associated With Universal Curriculum-Based Interventions in Early Childhood Education and Care Centers: A Systematic Review and Meta-analysis

**DOI:** 10.1001/jamanetworkopen.2018.5727

**Published:** 2018-12-07

**Authors:** Claire Blewitt, Matthew Fuller-Tyszkiewicz, Andrea Nolan, Heidi Bergmeier, David Vicary, Terry Huang, Paul McCabe, Tracey McKay, Helen Skouteris

**Affiliations:** 1 Monash Centre for Health Research and Implementation, School of Public Health and Preventive Medicine, Monash University, Clayton, Australia; 2 School of Psychology, Deakin University, Geelong, Australia; 3 Faculty of Arts and Education, Deakin University, Geelong, Australia; 4 Victoria Family & Community Services, Baptcare, Victoria, Australia; 5 Center for Systems and Community Design, School of Public Health, City University of New York, New York, New York; 6 School Psychologist Graduate Program, School Psychology Forum, Department of School Psychology, Counseling and Leadership, City University of New York, New York, New York; 7 Early Years Service, bestchance Child Family Care, Melbourne, Australia

## Abstract

**Importance:**

Social-emotional competence in early childhood influences long-term mental health and well-being. Interest in the potential to improve child health and educational outcomes through social and emotional learning (SEL) programs in early childhood education and care (ECEC) settings is increasing.

**Objective:**

To conduct a systematic review and meta-analysis of studies examining the social, emotional, and early learning outcomes associated with universal curriculum-based SEL programs delivered to children aged 2 to 6 years in center-based ECEC settings.

**Data Sources:**

Keyword searches of Education Resources Information Center (ERIC), MEDLINE Complete, PsycINFO, and Proquest Dissertations and Theses Global databases were conducted to identify all relevant studies published from January 1, 1995, through December 31, 2017.

**Study Selection:**

Studies included in this review examined universal curriculum-based SEL intervention delivered to children aged 2 to 6 years in a center-based ECEC setting. All assessed individual-level social and/or emotional skill after the SEL intervention and used an experimental or quasi-experimental design (ie, studies that did not or were not able to randomly allocate participants to intervention and control groups) with a control group.

**Data Extraction and Synthesis:**

A total of 13 035 records were screened, of which 362 were identified for full-text review. A systematic literature review was conducted on 79 studies. Multilevel random-effects meta-analyses were conducted on 63 eligible studies from October 2 through 18, 2018.

**Main Outcomes and Measures:**

Social competence, emotional competence, behavioral self-regulation, behavior and emotional challenges, and early learning outcomes.

**Results:**

This review identified 79 unique experimental or quasi-experimental studies evaluating the effect of SEL interventions on preschooler outcomes, including a total of 18 292 unique participants. Sixty-three studies were included in this meta-analysis. Compared with control participants, children in intervention conditions showed significant improvement in social competence (Cohen *d *[SE], 0.30; [0.06]; 95% CI, 0.18-0.42; *P* < .001), emotional competence (Cohen *d *[SE], 0.54 [0.16]; 95% CI, 0.22-0.86; *P* < .001), behavioral self-regulation (Cohen *d *[SE], 0.28 [0.09]; 95% CI, 0.11-0.46; *P* < .001), and early learning skills (Cohen *d *[SE], 0.18 [0.08]; 95% CI, 0.02-0.33; *P* = .03) and reduced behavioral and emotional challenges (Cohen *d *[SE], 0.19 [0.04]; 95% CI, 0.11-0.28; *P* < .001). Several variables appeared to moderate program outcomes, including intervention leader, type of assessment, informant, child age, and study quality.

**Conclusions and Relevance:**

According to results of this study, social and emotional learning programs appeared to deliver at a relatively low intensity may be an effective way to increase social competence, emotional competence, behavioral self-regulation, and early learning outcomes and reduce behavioral and emotional difficulties in children aged 2 to 6 years. Social and emotional learning programs appear to be particularly successful at increasing emotional knowledge, understanding, and regulation.

## Key Points

**Question:**

How effective are universal curriculum-based social and emotional learning programs delivered in early childhood education and care centers at improving children’s social and emotional development?

**Findings:**

A systematic review and meta-analysis of 79 unique studies with 18 292 unique participants found children exposed to a universal social and emotional learning intervention showed significant improvement in social competence, emotional competence, behavioral self-regulation, emotional and behavioral problems, and early learning outcomes compared with control participants.

**Meaning:**

Early childhood is a crucial period for children’s social, emotional, and cognitive development, and these findings highlight what appears to be benefit of social and emotional learning interventions for young children across developmental domains.

## Teaser

This systematic review and meta-analysis evaluates studies examining the social, emotional, and early learning outcomes associated with universal curriculum-based social and emotional learning programs delivered to children aged 2 to 6 years in center-based early childhood education and care settings.

## Introduction

The preschool period presents a unique opportunity to support children’s social and emotional development. During their formative years, children learn to understand and regulate emotion, attention, and behavior, equipping them to form prosocial relationships and engage in learning when they commence school.^1,2^ Difficulty navigating early social-emotional milestones can hinder a child’s emotional regulation, social behavior, and school readiness^3,4,5,6^ and lead to the development of mental health disorders.^7,8,9,10^

With an average of 78% of 3-year-old and 87% of 4-year-old children from 36 Organisation for Economic Co-operation and Development countries (27 European nations, United States, Canada, Australia, New Zealand, Chile, Japan, Israel, Korea, and Mexico) enrolled in early childhood or preprimary education,^11^ demand is growing from educators, researchers, and policy makers for evidence-based preventative and early-intervention early childhood education and care (ECEC) programs that target social, emotional, and behavioral outcomes for preschool children.^1,12,13^ Strengthening social and emotional competencies through teaching, modeling, and practice underpins social and emotional learning (SEL), defined by the Collaborative for Academic, Social, and Emotional Learning as the acquisition and application of knowledge and skills across 5 areas of social-emotional competence, including self-awareness, social awareness, self-management, relationship skills, and responsible decision making.^14^ Neuroscience research^15,16,17^ indicates SEL may have unique leverage for children aged 3 to 6 years when language and executive functions are rapidly developing; in addition, SEL intervention in preschool targets an age when children are especially receptive to external guidance and support.^18^

Several reviews have focused on the effects of SEL intervention in the preschool years. McCabe and Altamura^19^ revealed 10 intervention programs with demonstrated efficacy, but they also suggested further research was needed to identify the practices and approaches that a make substantive and lasting impression on social-emotional competence. Schindler et al^20^ found that SEL programs led to greater reduction in externalizing behavior compared with those without an explicit focus on SEL. In contrast, Sabey et al^21^ found that SEL interventions (11 of 26 studies they reviewed) demonstrated weaker effects and lower research quality compared with programs focusing on behavior, coping, or other social-emotional skills. Bierman and Motamedi^18^ identified only 2 preschool-based SEL programs with a robust evidence base (Promoting Alternative Thinking Strategies [PATHS] and the Incredible Years Teaching Program) and 3 that showed promise (Tools of the Mind, I Can Problem Solve, and Al’s Pal’s: Kids Making Healthy Choices). Another recent review reported the small-to-medium effects from SEL intervention in early childhood were encouraging, but highlighted the challenge in comparing programs that are based on different theoretical frameworks, target different skills, and often use different outcome measures.^22^

Research that unpacks the active ingredients of successful SEL approaches is needed.^23^ Hence, the objective of this review was to address the following research questions: (1) What social, emotional, behavioral, and early learning outcomes have been achieved by universal curriculum-based SEL interventions implemented in ECEC settings? (2) What program-level characteristics are associated with positive outcomes? and (3) What are the methodologic limitations of research investigating the outcomes achieved by curriculum-based SEL interventions in ECEC settings? We conclude with recommendations for future research.

## Methods

### Search Strategy and Study Selection

This systematic review and meta-analysis was conducted in accordance with the recommendations and standards set by the Preferred Reporting Items for Systematic Reviews and Meta-analyses (PRISMA) reporting guideline.

Published, peer-reviewed reports were sourced through computerized database searches of Education Resources Information Center (ERIC), MEDLINE Complete, and PsycINFO (January 1, 1995, through December 31, 2017). No language limits were applied. The key terms included in the database searches and an example search strategy are provided in the eFigure in the Supplement. These searches identified 10 189 articles after the removal of duplicates. A manual search of references cited in selected reports and relevant reviews and meta-analyses of intervention programs targeting early childhood social and emotional development was undertaken, and suitable reports were included. To address possible file-drawer effects,^24^ a systematic search of dissertations through the Proquest Dissertations and Theses Global database was conducted. Abstracts were searched using combinations of terms, with a further 2846 reports identified, resulting in a total of 13 035 reports screened.

Studies met inclusion criteria if (1) they delivered a universal curriculum-based SEL program to children aged 2 to 6 years in a center-based ECEC setting (ie, included explicit teaching of SEL skills); (2) the primary stated purpose of the SEL program was to increase children’s social-emotional skill development; (3) they assessed individual-level social, emotional, behavioral, and/or learning skills after the SEL intervention; and (4) they used an experimental or quasi-experimental design (ie, studies that did not or were not able to randomly allocate participants to intervention and control groups) with a control group. All titles and abstracts were screened for possible inclusion by 1 author (C.B.). A trained research assistant independently coscreened 10% (n = 1300) of the titles and abstracts; agreement for the inclusion of articles to be read in full was 100%.

### Data Extraction

Extracted data included (1) publication status; (2) sample size; (3) design; (4) whether pretest measurements were recorded; (5) age of children; (6) sex distribution; (7) nationality of children; (8) child’s socioeconomic status; (9) age of SEL program; (10) frequency and duration of sessions/lessons; (11) whether the intervention was teacher, specialist, or researcher led; (12) whether the intervention was delivered to the classroom or a small group; (13) whether the intervention included parental involvement; (14) informant (parent, teacher, or other); (15) whether outcome reflected skill acquisition, assessed through structured test or task; and (16) whether implementation fidelity was considered. To ensure accuracy and reliability, 2 independent reviewers (including C.B.) coded 70% of studies, with any discrepancies resolved by consensus reached after discussion.

The child outcomes from each study were assigned a category, informed by 4 social-emotional subdomains and constructs identified by Jones et al^25^ and the Federal Interagency Forum on Child and Family Statistics,^26^ including social competence, emotional competence, behavior/emotional challenges, and behavioral self-regulation. A fifth category reflecting early learning outcomes was also included with measures of oral language, vocabulary, early literacy, and math ability. This categorization reflects current knowledge of early childhood social-emotional development and offers a relevant framework to understand and compare SEL intervention across outcomes.

In the instance where an outcome could be allocated to more than 1 category, we assigned the category that most closely matched the description of the measure. To determine the quality of included studies, each study was assessed against the Effective Public Health Practice Project quality assessment tool for quantitative studies with respect to selection bias, study design, confounders, blinding, data collection methods, withdrawals, dropouts, intervention integrity, and analyses.^27^

### Calculation of Effect Sizes

For each outcome, the standardized mean difference (Cohen *d*) was calculated by dividing the difference between posttest SEL scores of the control group and intervention group by the pooled SD.^28^ The first measurement recorded after program completion has been included in the analyses. Many studies provided sufficient data to calculate the standardized mean difference between the intervention and control groups before the intervention. To account for potential differences at baseline, this pretest effect size was subtracted from the postintervention effect where available. According to Cohen,^29^ a value of 0.2 is considered a small effect; 0.5, a moderate effect; and 0.8, a large effect. Effect size measures were allocated a positive sign if the data indicated the intervention had higher, more positive scores on the variable of interest relative to the control group. Some studies reported the total or composite score in addition to subscale scores on standardized tests. Where subscale scores that were meaningful in the context of this review were included in the calculation of total or composite scale scores, we selected only the subscale score to avoid duplicate effects.

When the data needed to compute the standardized mean difference between posttest intervention and control group scores were not available within published studies, we requested these data from the corresponding author. If we were unable to contact the corresponding author or the study authors were unable to provide such data, the report was retained in the systematic review but excluded from the meta-analysis (Figure).

**Figure.**
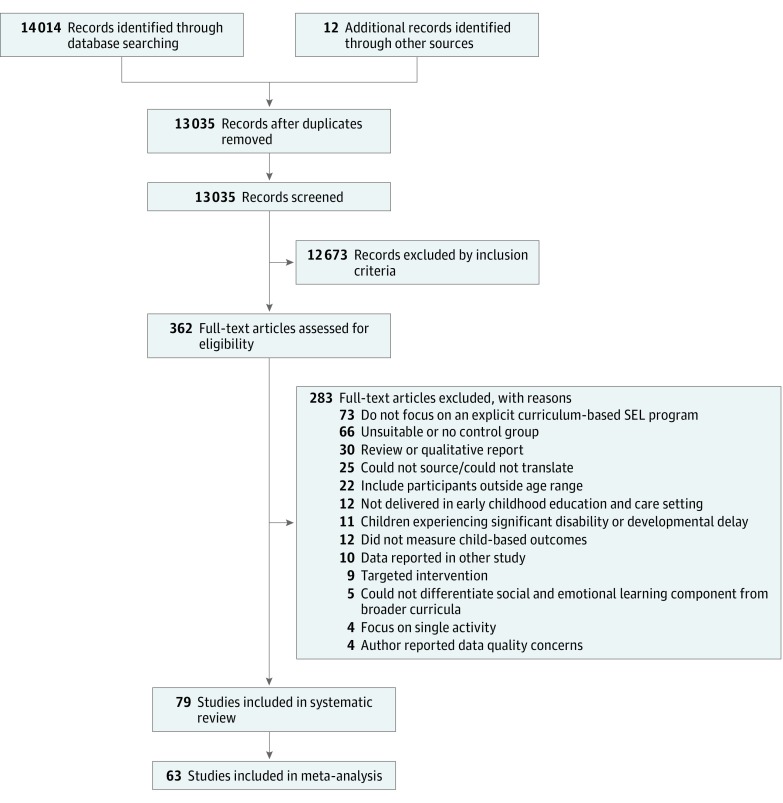
Selection of Studies Included in the Meta-analysis Identification SEL indicates social and emotional learning.

### Statistical Analysis

Data were analyzed from October 2 through 18, 2018. Several reports included in this study had multiple estimates of the same effect. Given that these effect sizes are drawn from the same sample of children, they violate the assumption of statistical independence.^30^ To account for the nesting of effect sizes within studies, a multilevel model framework was used to determine (1) the mean effect size across all studies and (2) the mean effect size across each outcome category while controlling for nonindependence due to multiple estimates within the same study.^31^ The heterogeneity of effect sizes across studies was assessed using the intraclass correlation (ICC) and *I*^2^ and τ^2^ tests. In addition, the significance of the heterogeneity of each group of effect sizes was examined with the Q statistic, where a significant Q value indicates studies are not derived from a common population.

To examine the moderation effect of study-level characteristics, a metaregression was undertaken when ICC values were greater than 0.25 (25% of variance explained by across-study variation in effect sizes). Where heterogeneity of effect sizes was detected, each moderator was examined separately to identify the characteristics that might explain these differences. Where multiple moderators were shown to be significant, they were modeled simultaneously to address potential confounding. Only significant moderators from this step were included in the final model. Statistical significance was set at 2-tailed *P* < .05. All analyses were performed using the metafor package^32^ in RStudio (version 1.1.383).

### Publication Bias

We addressed the potential for publication bias in 3 ways. First, we included unpublished dissertations as described above. Second, we included publication status as a moderator to determine whether a significant difference between outcomes reported in published studies and dissertations existed. Third, we applied the Egger regression test^33^ to test for publication bias. When the intercept of this test deviates significantly from zero (at *P* = .10),^33^ the overall association between the precision and size of studies is considered asymmetrical, with potential for bias.

## Results

### Systematic Review Results

The Figure shows a flow diagram of our systematic review and meta-analysis conducted in accordance with the PRISMA guidelines. Seventy-nine unique studies were deemed relevant for this review, including a total 18 292 unique participants. Sixty-three studies were available for the meta-analysis. The pooled sample characteristics for all studies and the characteristics within each domain of social-emotional functioning are provided in Table 1 and detailed further in eTable 1 in the Supplement.^34,35,36,37,38,39,40,41,42,43,44,45,46,47,48,49,50,51,52,53,54,55,56,57,58,59,60,61,62,63,64,65,66,67,68,69,70,71,72,73,74,75,76,77,78,79,80,81,82,83,84,85,86,87,88,89,90,91,92,93,94,95,96,97,98,99,100,101,102,103,104,105,106,107,108,109,110,111,112^

**Table 1.** Descriptive Characteristics of 79 Studies Examining SEL in ECEC Settings

Characteristics	Studies, No. (%) of Participants^a^					
	All (n = 79)	Social Competence (n = 61)	Emotional Competence (n = 41)	Problem Behaviors and Emotions (n = 58)	Behavioral Self-regulation (n = 16)	Early Learning Outcomes (n = 16)
Geographic location						
Africa	1 (1.3)	1 (1.6)	0	1 (1.7)	0	0
Australia	4 (5.1)	4 (6.6)	2 (4.9)	4 (6.9)	1 (6.2)	1 (6.2)
Europe	21 (26.6)	17 (27.9)	15 (36.6)	13 (22.4)	0	2 (12.5)
Middle East	1 (1.3)	1 (1.6)	0	0	0	0
North America	51 (64.6)	38 (62.3)	23 (56.1)	40 (69.0)	15 (93.8)	13 (81.3)
South America	1 (1.3)	0	1 (2.4)	0	0	0
Date of report						
1995-2007	21 (26.6)	17 (27.9)	7 (17.1)	17 (29.3)	10 (62.5)	3 (18.8)
2008-2012	30 (38.0)	21 (34.4)	14 (34.1)	21 (36.2)	3 (18.8)	7 (43.8)
2013-2017	28 (35.4)	23 (37.7)	20 (48.8)	20 (34.5)	3 (18.8)	6 (37.5)
Publication status						
Peer-reviewed journal^b^	68 (86.1)	55 (90.2)	36 (87.8)	49 (84.5)	15 (93.8)	15 (93.8)
Dissertation	11 (13.9)	6 (9.8)	5 (12.2)	9 (15.5)	1 (6.2)	1 (6.2)
Sample size						
≤100	33 (41.8)	26 (42.6)	19 (46.3)	22 (37.9)	5 (31.3)	7 (43.8)
101-200	18 (22.8)	11 (18.0)	8 (19.5)	14 (24.1)	4 (25.0)	2 (12.5)
201-300	12 (15.2)	9 (14.8)	4 (9.8)	9 (15.5)	3 (18.8)	2 (12.5)
301-500	10 (12.7)	9 (14.8)	5 (12.2)	9 (15.5)	4 (25.0)	2 (12.5)
>500	6 (7.6)	6 (9.8)	5 (12.2)	4 (6.9)	0	3 (18.8)
Age of children, y						
≤3	5 (6.3)	5 (8.2)	4 (9.8)	5 (8.6)	0	2 (12.5)
3-5	46 (58.2)	35 (57.4)	23 (56.1)	35 (60.3)	12 (75.0)	10 (62.5)
>5	25 (31.6)	18 (29.5)	12 (29.3)	15 (25.9)	4 (25.0)	4 (25.0)
Described as preschool or kindergarten age, or across age ranges	3 (3.8)	3 (4.9)	2 (4.9)	3 (5.2)	0	0
SES of sample						
Low	30 (38.0)	24 (39.3)	17 (41.5)	26 (44.8)	10 (62.5)	8 (50.0)
Middle or high	14 (17.7)	9 (14.8)	7 (17.1)	8 (13.8)	2 (12.5)	4 (25.0)
Mixed	12 (15.2)	8 (13.1)	4 (9.8)	10 (17.2)	2 (12.5)	2 (12.5)
Not reported	23 (29.1)	20 (32.8)	13 (31.7)	14 (24.1)	2 (12.5)	2 (12.5)
Intervention leader						
Teacher	53 (67.1)	46 (75.4)	28 (68.3)	44 (75.9)	12 (75.0)	10 (62.5)
Specialist	22 (27.8)	13 (21.3)	11 (26.8)	12 (20.7)	4 (25.0)	6 (37.5)
Not specified	4 (5.1)	2 (3.3)	2 (4.9)	2 (3.4)	0	0
Program duration, wk						
<6	7 (8.9)	5 (8.25)	5 (12.2)	5 (8.6)	0	1 (6.2)
6-12	27 (34.2)	17 (27.9)	12 (29.3)	17 (29.3)	5 (31.3)	7 (43.8)
12-24	26 (32.9)	22 (36.1)	14 (34.1)	20 (34.5)	6 (37.5)	2 (12.5)
>24	17 (21.5)	14 (23.0)	10 (24.4)	14 (24.1)	4 (25.0)	6 (37.5)
Not reported	2 (2.5)	3 (4.9)	0	2 (3.4)	1 (6.3)	0
Instruction time, min/wk						
≤30	14 (17.7)	11 (18.0)	8 (19.5)	9 (15.5)	4 (25.0)	2 (12.5)
31-60	29 (36.7)	22 (36.1)	15 (36.6)	21 (36.2)	3 (18.8)	5 (31.2)
60-120	15 (19.0)	12 (19.7)	9 (22.0)	9 (15.5)	2 (12.5)	2 (12.5)
>120	5 (6.3)	3 (4.9)	1 (2.4)	4 (6.9)	2 (12.5)	2 (12.5)
Not reported	16 (20.3)	13 (21.3)	8 (19.5)	15 (25.9)	5 (31.2)	5 (31.2)
Attempted to engage caregiver						
Yes	32 (40.5)	28 (45.9)	16 (39.0)	26 (44.8)	7 (43.8)	4 (25.0)
No or not clear	47 (59.5)	33 (54.1)	25 (60.9)	32 (55.2)	9 (56.3)	12 (75.0)
Informant						
Parent report	19 (24.1)	18 (29.5)	29 (70.7)	18 (31.0)	6 (37.5)	2 (12.5)
Teacher report	59 (74.7)	49 (80.3)	29 (70.7)	50 (86.2)	14 (87.5)	11 (68.8)
Observed	46 (58.2)	32 (54.5)	11 (26.8)	29 (50.0)	10 (62.5)	14 (87.5)
Authors considered implementation fidelity						
Yes	48 (60.8)	37 (60.7)	24 (58.5)	38 (65.5)	11 (68.8)	12 (75.0)
No or not clear	31 (39.2)	24 (39.3)	17 (41.5)	20 (34.5)	5 (31.2)	4 (25.0)

Abbreviations: ECEC, early childhood education and care; SEL, social and emotional learning; SES, socioeconomic status.

^a^ Percentages have been rounded and may not total 100.

^b^ Includes 1 published government report.

We found variability in study quality. Twelve studies^41,44,54,67,73,76,82,83,87,106,109,111^ (16.0%) were rated as high quality; 33 studies^37,40,45,46,47,48,50,56,57,58,59,60,63,64,66,74,78,80,85,86,90,91,92,93,96,97,99,103,104,105,108,110,112^ (44.0%), moderate quality; and 30 studies^34,36,38,39,42,43,49,51,52,53,55,61,62,65,68,69,71,72,75,79,84,88,89,94,95,98,100,101,102,107^ (40.0%), poor quality. Four non-English studies^35,70,77,81^ were excluded from the quality assessment. Most studies were downgraded owing to the lack of blinding, which can be difficult to achieve in educational research. Lower-quality studies were also less likely to report and control for confounding variables in their analyses. The constructs assessed within each domain of social-emotional development and the measures used are provided in eTable 3 in the Supplement. Several studies^37,44,46,51,61,74,77,83,86,88,89,91,95,113^ collected follow-up data at least 1 month after the intervention concluded and reported sustainability of the program effect over time.

### Universal SEL Approaches

Fifty-one SEL programs were examined across the 79 studies (eTable 2 in the Supplement). Interventions drew on overlapping theories of child development and shared a common goal to increase children’s social and emotional skills through explicit and active instruction, modeling, opportunity for practice, and reinforcement, typically using classroom routines and activities (eg, circle time, small-group sessions, and play) and developmentally appropriate teaching methods (eg, storytelling, singing, role play, and puppetry). They differed, however, in their underlying theory of change; programs targeted varying mediating pathways to social and emotional competence,^82^ with some addressing a broad and interrelated set of cognitive, behavioral, and affective skills and others addressing focal skills that encourage specific competencies such as mindfulness, coping and resilience, social problem solving, and conversational strategies (eTable 2 in the Supplement).

### Meta-analysis Results

#### Overall Outcomes of Program Participation

The overall weighted mean (SE) effect size for all 391 effects was Cohen *d* = 0.38 (0.07) (95% CI, 0.24-0.51; *P* < .001). The results from the unconditional models and metaregression are provided in Table 2 and Table 3, respectively. In the overall model, the proportion of variance in effect size between studies determined by the ICC was 84.5%, and several significant moderators were identified. Improved outcomes were observed for older children (unstandardized β [B] = 0.13; SE, 0.06; *P* = 0.03) and in programs delivered by a specialist or researcher rather than the classroom teacher (B = −0.28; SE, 0.14; *P* = .04). Assessment of child functioning based on the parent report suggested less improvement after program participation compared with measures completed by teachers, observers, or researchers (B = −0.23; SE, 0.05; *P* < .001). Furthermore, children displayed greater improvement in skill-based measures that were assessed in a test situation or structured task, compared with teacher, parent, or observer ratings of behavior (B = 0.20; SE, 0.05; *P* < .001). Higher-quality studies (those rated moderate or strong) were associated with lower effect sizes compared with lower-quality studies (B = −0.33; SE, 0.15; *P* = .03). When all significant variables were included in the model, parent informant (B = −0.19; SE, 0.05; *P* < .001) and skill-based measures (B = 0.15; SE, 0.05; *P* = .002) showed a significant unique effect, whereas intervention leader (B = −0.25; SE, 0.15; *P* = .09) and study quality (B = −0.32; SE, 0.16; *P* = .05) did not. Parent informant and skills-based measures remained significant unique moderators in step 3 of the model (Table 3).

**Table 2.** Unconditional Model Estimating Effect Sizes for Measures of Social-Emotional Functioning

Outcome Category	No. of Effects	Cohen *d* (SE) [95% CI]	*z* Value	*I*^2^ Value		τ^2^ Value		*Q* Statistic^a^	ICC
				Between	Within	Between	Within		
All	391	0.38 (0.07) [0.24-0.51]	5.33^a^	78.38	14.34	0.29	0.05	2422.60	0.85
Social competence	115	0.30 (0.06) [0.18-0.42]	4.93^a^	59.02	26.58	0.11	0.05	782.33	0.69
Emotional competence	54	0.54 (0.16) [0.22 -0.86]	3.33^a^	59.71	36.83	0.54	0.33	714.42	0.62
Problem behaviors and emotions	170	0.19 (0.04) [0.11-0.28]	4.43^a^	56.64	18.63	0.06	0.02	676.79	0.75
Self-regulation	16	0.28 (0.09) [0.11 -0.46]	3.12^a^	20.54	58.88	0.02	0.07	83.82	0.25
Early learning outcomes	36	0.18 (0.08) [0.02-0.33]	2.18^b^	65.63	14.33	0.07	0.01	111.34	0.82

Abbreviation: ICC, intraclass correlation.

^a^ *P* < .001.

^b^ *P* < .05.

**Table 3.** Metaregression Predicting Effect Sizes for Measures of Social-Emotional Functioning

Moderators for Each Category	Analysis								
	Single Moderators			All Significant Moderators			Only Significant Moderators		
	B^a^ (SE)	*z* Value	*P* Value	B^a^ (SE)	*z* Value	*P* Value	B^a^ (SE)	*z* Value	*P* Value
**All Outcomes**									
Publication status	0.05 (0.19)	0.25	.80	NA	NA	NA	NA	NA	NA
Program’s age	0.00 (0.01)	−0.14	.89	NA	NA	NA	NA	NA	NA
Randomization	−0.15 (0.14)	−1.09	.28	NA	NA	NA	NA	NA	NA
Pretest	−0.12 (0.08)	−1.40	.16	NA	NA	NA	NA	NA	NA
Age of children	0.13 (0.06)	2.19	.03	0.03 (0.07)	0.50	.62	NA	NA	NA
Sex	0.00 (0.00)	1.50	.14	NA	NA	NA	NA	NA	NA
SES	−0.12 (0.14)	−0.85	.40	NA	NA	NA	NA	NA	NA
Instruction time, min/wk	0.00 (0.00)	1.08	.28	NA	NA	NA	NA	NA	NA
Length of program, wk	−0.00 (0.01)	−0.47	.64	NA	NA	NA	NA	NA	NA
Intervention leader^b^	−0.28 (0.14)	−2.02	.04	−0.25 (0.15)	−1.70	.09	NA	NA	NA
Mode of delivery^c^	−0.30 (0.20)	−1.49	.14	NA	NA	NA	NA	NA	NA
Parental involvement	0.11 (0.15)	0.74	.46	NA	NA	NA	NA	NA	NA
Parent informant	−0.23 (0.05)	−4.25	<.001	−0.19 (0.05)	−3.57	<.001	−0.19 (0.06)	−3.34	<.001
Teacher informant	−0.02 (0.04)	−0.39	.70	NA	NA	NA	NA	NA	NA
Skills-based measure	0.20 (0.05)	4.22	<.001	0.15 (0.05)	3.13	.002	0.16 (0.05)	3.31	.001
Study quality^d^	−0.33 (0.15)	−2.18	.03	−0.32 (0.16)	−1.92	.05	NA	NA	NA
**Social Competence**									
Publication status	−0.05 (0.20)	0.26	.80	NA	NA	NA	NA	NA	NA
Program’s age	0.00 (0.01)	−0.27	.79	NA	NA	NA	NA	NA	NA
Randomization	−0.02 (0.13)	−0.15	.88	NA	NA	NA	NA	NA	NA
Pretest	−0.19 (0.15)	−1.27	.22	NA	NA	NA	NA	NA	NA
Age of children	0.10 (0.05)	2.06	.04	0.07 (0.04)	1.65	.10	NA	NA	NA
Sex	0.00 (0.00)	1.56	.13	NA	NA	NA	NA	NA	NA
SES	−0.16 (0.12)	−1.28	.20	NA	NA	NA	NA	NA	NA
Instruction time, min/wk	0.00 (0.00)	1.20	.23	NA	NA	NA	NA	NA	NA
Length of program, wk	0.00 (0.01)	−0.53	.60	NA	NA	NA	NA	NA	NA
Intervention leader^b^	−0.43 (0.13)	−3.28	.001	−0.35 (0.10)	−3.61	<.001	−0.38 (0.13)	−3.10	.002^e^
Mode of delivery^c^	−0.31 (0.19)	−1.63	.10	NA	NA	NA	NA	NA	NA
Parental involvement	0.04 (0.12)	0.39	.70	NA	NA	NA	NA	NA	NA
Parent informant	−0.13 (0.10)	−1.38	.17	NA	NA	NA	NA	NA	NA
Teacher informant	−0.15 (0.08)	−2.00	.05	NA	NA	NA	NA	NA	NA
Skills-based measure	0.35 (0.10)	3.51	<.001	0.27 (0.10)	2.72	.006	0.32 (0.10)	3.33	.002^e^
Study quality^d^	−0.15 (0.13)	−1.13	.26	NA	NA	NA	NA	NA	NA
**Emotional Competence**									
Publication status	0.19 (0.47)	0.41	.68	NA	NA	NA	NA	NA	NA
Program’s age	−0.02 (0.03)	−0.64	.53	NA	NA	NA	NA	NA	NA
Randomization	−0.15 (0.36)	−0.42	.68	NA	NA	NA	NA	NA	NA
Pretest	0.03 (0.34)	0.07	.94	NA	NA	NA	NA	NA	NA
Age of children	0.15 (0.14)	1.08	.28	NA	NA	NA	NA	NA	NA
Sex	−0.02 (0.05)	−0.34	.73	NA	NA	NA	NA	NA	NA
SES	−0.27 (0.33)	−0.83	.41	NA	NA	NA	NA	NA	NA
Instruction time, min/wk	0.00 (0.01)	0.65	.52	NA	NA	NA	NA	NA	NA
Length of program, wk	−0.02 (0.02)	−1.18	.24	NA	NA	NA	NA	NA	NA
Intervention leader^b^	−0.20 (0.36)	−0.55	.58	NA	NA	NA	NA	NA	NA
Mode of delivery^c^	−0.52 (0.46)	−1.13	.26	NA	NA	NA	NA	NA	NA
Parental involvement	0.17 (0.34)	0.49	.62	NA	NA	NA	NA	NA	NA
Parent informant	−0.25 (0.38)	−0.65	.51	NA	NA	NA	NA	NA	NA
Teacher informant	−0.30 (0.27)	−1.12	.27	NA	NA	NA	NA	NA	NA
Skills-based measure	0.44 (0.24)	1.84	.07	NA	NA	NA	NA	NA	NA
Study quality^d^	−0.80 (0.32)	−2.48	.01	NA	NA	NA	NA	NA	NA
**Problem Behaviors and Emotions**									
Publication status	−0.02 (0.11)	−0.18	.85	NA	NA	NA	NA	NA	NA
Program’s age	0.01 (0.01)	0.90	.37	NA	NA	NA	NA	NA	NA
Randomization	−0.13 (0.09)	1.39	.17	NA	NA	NA	NA	NA	NA
Pretest	−0.14 (0.09)	−1.23	.22	NA	NA	NA	NA	NA	NA
Age of children	0.04 (0.05)	0.81	.42	NA	NA	NA	NA	NA	NA
Sex	0.00 (0.00)	1.12	.26	NA	NA	NA	NA	NA	NA
SES	−0.06 (0.09)	−0.70	.48	NA	NA	NA	NA	NA	NA
Instruction time, min/wk	0.000 (0.00)	−0.12	.91	NA	NA	NA	NA	NA	NA
Length of program, wk	0.000 (0.00)	−0.02	.98	NA	NA	NA	NA	NA	NA
Intervention leader^b^	−0.23 (0.10)	−2.37	.02	−0.22 (0.10)	−2.20	.03	NA	NA	NA
Mode of delivery^c^	−0.12 (0.14)	−0.84	.40	NA	NA	NA	NA	NA	NA
Parental involvement	0.05 (0.09)	0.62	.54	NA	NA	NA	NA	NA	NA
Parent informant	−0.23 (0.06)	−4.09	<.001	−0.23 (0.06)	−4.00	<.001	NA	NA	NA
Teacher informant	0.10 (0.05)	1.90	.06	NA	NA	NA	NA	NA	NA
Skills-based measure	0.08 (0.10)	0.84	.40	NA	NA	NA	NA	NA	NA
Study quality^d^	−0.06 (0.10)	−0.58	.56	NA	NA	NA	NA	NA	NA
**Early Learning Outcomes**									
Publication status	0.49 (0.28)	**1.77**	.08	NA	NA	NA	NA	NA	NA
Program’s age	0.00 (0.01)	−0.23	.82	NA	NA	NA	NA	NA	NA
Randomization	−0.49 (0.28)	−1.77	.08	NA	NA	NA	NA	NA	NA
Pretest	−0.02 (0.14)	−0.17	.87	NA	NA	NA	NA	NA	NA
Age of children	−0.04 (0.09)	−0.51	.61	NA	NA	NA	NA	NA	NA
Sex	0.01 (0.02)	0.44	.66	NA	NA	NA	NA	NA	NA
SES	−0.30 (0.14)	−2.18	.03	−0.21 (0.145)	−1.51	.13	NA	NA	NA
Instruction time, min/wk	0.00 (0.00)	0.36	.72	NA	NA	NA	NA	NA	NA
Length of program, wk	0.00 (0.01)	−0.20	.84	NA	NA	NA	NA	NA	NA
Intervention leader^b^	−0.25 (0.16)	1.58	.11	NA	NA	NA	NA	NA	NA
Mode of delivery^c^	−0.35 (0.16)	−2.16	.03	−0.26 (0.17)	−1.58	.12	NA	NA	NA
Parental involvement	−0.05 (0.18)	−0.30	.77	NA	NA	NA	NA	NA	NA
Parent informant	NA	NA	NA	NA	NA	NA	NA	NA	NA
Teacher informant	−0.49 (0.29)	1.67	.10	NA	NA	NA	NA	NA	NA
Skills-based measure	0.15 (0.20)	0.74	.46	NA	NA	NA	NA	NA	NA
Study quality^d^	−0.49 (0.30)	1.67	.10	0.14 (0.21)	0.67	.50	NA	NA	NA

Abbreviations: NA, not applicable; SES, socioeconomic status.

^a^ Unstandardized β.

^b^ Includes specialist, researcher, or teacher.

^c^ Includes small group or classroom.

^d^ Includes low, medium, or high.

#### Social Competence

The weighted mean (SE) effect size in the social competence category was Cohen *d* = 0.30 (0.06) (95% CI, 0.18-0.42; *P* < 001). The test of heterogeneity showed variability across effect sizes (ICC = 0.69). The following were significant moderators when the data was examined in separate analyses: child age (B = 0.10; SE, 0.05; *P* = .04), intervention leader (B = −0.43; SE, 0.13; *P* < .001), and skills-based assessment (B = 0.35; SE, 0.10; *P* < .001), with mode of delivery (B = −0.31; SE, 0.19; *P* = .10) and teacher informant (B = −0.15; SE, 0.08; *P* = .05) meaningful but not significant. In a model including all significant variables, intervention leader (B = −0.35; SE, 0.10; *P* < .001) and skills-based measures (B = 0.27; SE, 0.10; *P* = .006) were significant unique moderators. These moderators remained significant when modeled simultaneously.

#### Emotional Competence

A medium to large effect on measures of emotional competence was found for the mean of 54 effect sizes (Cohen *d* [SE], 0.54 [0.16]; 95% CI, 0.22-0.86; *P* < 001). The proportion of variance determined by the ICC of 61.8% suggests moderator analyses were appropriate for this domain. Only 1 moderator reached significance; lower effect sizes were associated with higher-quality studies (B = −0.80; SE, 0.32; *P* = .01). Assessment with skill-based measure reached borderline significance (B = 0.44; SE, 0.24; *P* = .07).

#### Behavioral and Emotional Difficulties

The weighted mean effect size in this category was small (Cohen *d *[SE], 0.19 [0.04]; 95% CI, 0.11-0.28; *P* < .001), and the test of heterogeneity showed significant variability across effects (ICC = 0.75). The metaregression indicated specialist- or researcher-led programs (B = −0.23; SE, 0.10; *P* = .02) resulted in stronger effect sizes. Parent assessment of child behavior suggested less improvement (B = −0.23; SE, 0.06; *P* < .001), whereas greater improvement based on teacher report was identified (B = 0.10; SE, 0.05; *P* = .06); however, this did not reach significance. When significant moderators were analyzed together, parent informant (B = −0.23; SE, 0.06; *P* < .001) and intervention leader (B = −0.22; SE, 0.10; *P* = .03) remained significant.

#### Self-regulation

Sixteen effects within 13 studies^44,46,54,64,65,67,71,78,80,91,96,106,112^ included a measure of behavioral self-regulation with a mean (SE) effect size of 0.28 (0.09) (95% CI, 0.11-0.46; *P* < .001). Evidence of substantial heterogeneity in effect size requiring metaregression was not evident in this category (ICC = 0.25).

#### Early Learning Outcomes

Overall, program participation showed a small but significant importance for early learning outcomes (Cohen *d *[SE], 0.18 [0.08]; 95% CI, 0.02-0.33; *P* = .03). The ICC of 0.82 suggests moderator analyses were suitable for this category. Programs that included small-group and individual teaching practices (B = −0.35; SE, 0.16; *P* = .03) were associated with larger effect sizes. The SEL programs did not appear as effective on learning outcomes for children from low socioeconomic backgrounds (B = −0.30; SE, 0.14; *P* = .03). Higher-quality studies reported lower effects (B = −0.49; SE, 0.30; *P* = .10), although this did not reach significance. Moderators did not reach significance when combined in a single model.

### Publication Bias

No significant asymmetry was detected in the overall data set (intercept = −0.01; SE, 0.10; *P* = .89), social competencies (intercept = 0.08; SE, 0.09; *P* = .37), emotional competencies (intercept = −0.01; SE, 0.23; *P* = .98), problem behaviors (intercept = 0.09; SE, 0.07; *P* = .23), behavioral self-regulation (intercept = 0.37; SE, 0.13; *P* = 004), or early learning outcomes (intercept = 0.04; SE, 0.12; *P* = .76). This result could indicate some degree of publication bias, or the tendency for smaller studies, which may be less rigorous, to be associated with larger effect sizes. Importantly however, publication status was examined as a moderator in the overall model and for each category, with no significant differences between published and unpublished studies found.

## Discussion

### What Outcomes Have Been Achieved by Curriculum-Based SEL Interventions Implemented in ECEC Settings?

Extensive research supports the efficacy and effectiveness of school-based SEL programs among older children and adolescents.^114^ The findings of this review indicate that universal SEL programs delivered to preschool-aged children offer benefit across a range of social-emotional domains that underpin healthy development. Participation led to significant improvements in social competence, emotional competence, self-regulation, and early learning skills and decreased behavioral and emotional difficulties.

The largest effect occurred for measures of emotional competence. Children who can understand and regulate their emotions are able to show empathy, navigate social friendships, and develop prosocial relationships. Research suggests that emotional competence in early childhood contributes to social competence concurrently and later in kindergarten,^115^ and emotional knowledge has been shown to be associated with social behavior and academic competence in later childhood.^116^ Therefore, encouraging children’s emotional skills through SEL intervention in the preschool years may have ongoing health and well-being benefits. Program outcome was not as pronounced for social competence or self-regulated behavior. This finding is consistent with reviews of social skills training that report stronger association with proximal factors (eg, child skill) than distal outcomes (eg, child behavior).^117^

Our findings suggest that early childhood SEL programs may have a smaller role in challenging behavior and emotions. After skills training, children may need time to practice and integrate learned behaviors into their behavior system before others will notice a change, a phenomenon known as the sleeper effect.^117^ However, most of the studies that included a measure of challenging behavior did not report follow-up data, and it is therefore difficult to determine whether this sleeper effect occurred. Studies examining universal preventive programs often fail to identify improvement in externalizing problems.^54,118,119^ This outcome may be influenced by limited measures available to assess behavioral problems in young children.^120^ Moreover, a number of socioecological factors may contribute to the development and maintenance of problematic behaviors and emotions. More intensive parenting modules within SEL interventions might improve outcomes in this domain; further research is needed.

### What Program Characteristics Are Associated With Positive Outcomes?

Programs delivered by facilitators, specialists, or researchers appeared more effective than those delivered by the classroom teacher, although the included studies did not consistently report teacher qualifications and experience, and therefore we could not ascertain whether and how educator differences influenced results. Han et al^64^ suggest educators require in-depth training, personal development, and performance feedback to support the introduction and maintenance of complex classroom interventions. Examination of the teacher training provided by SEL programs was outside the scope of this review; however, professional development varied in terms of methods, length, and ongoing support, which may have influenced teacher capacity to deliver programs with high fidelity.

Parents reported less improvement in their child after the intervention compared with the classroom teacher or an independent observer, which may indicate the possibility of bias owing to teacher expectations. Authors discussed the challenges in engaging parents in the SEL intervention programs. School-based intervention research has found that when parents are not involved in the program, effects may remain specific to the classroom.^121^ Furthermore, it is known that more intensive models that combine parent and teacher training lead to stronger outcomes that last over time.^122^ Continued efforts to understand the barriers to parental involvement and design home-based modules that complement work within the classroom appears warranted.

Studies reported a small but significant benefit for older children. The skills that underpin SEL (eg, perspective taking, organized thinking, reasoning, goal setting, attention, motivation, and self-regulated behavior) rely on executive regulatory systems^15,16,17^ that are shaped by biological and behavioral development. Older preschoolers may be equipped to glean more from these programs owing to maturation and experience, particularly with regard to social competencies. Finally, program’s age did not appear to moderate outcomes, suggesting recent programmatic efforts have not led to additional improvement above those programs designed in previous decades.

### Limitations

With the exception of a small number of randomized clinical trials, studies were constrained by sample size, the level of randomization possible in a classroom setting, reliance on teacher report of child outcomes, and limited engagement with parents. Larger trials with ethnically and socioeconomically diverse children will allow researchers to account for the effects of nesting of students within schools and better understand the extent of intervention outcomes.

Teacher and parent reports of child behavior and competencies provide an important perspective. However, the addition of objective assessment by raters blind to condition would lend credibility to the findings. In addition, it is imperative that researchers provide robust fidelity data to determine whether changes result from the intervention effect or a flaw in delivery.

Further exploration of the benefits of SEL intervention for children experiencing vulnerability is also needed. Studies varied in how they conceptualized and measured indices of risk. Closer examination of the outcomes for children most in need of intervention and the factors that influence whether these children access SEL programs in ECEC settings may assist professionals to reach children who are most likely to benefit from participation.

The differences in study outcomes may be influenced by the differing measures of social-emotional dimensions and constructs. Continued attention toward understanding the various pathways by which SEL interventions lead to specific developmental outcomes will allow programmers to target the skills and knowledge most likely to influence positive trajectories. We captured only explicit, curriculum-based SEL approaches. It is similarly important to examine and compare the benefit of implicit models that encourage educators to integrate SEL into everyday practices and core pedagogy. Further work is also needed to support teacher-led implementation of universal approaches. Closer examination of the professional development models available to educators and their effect on educator behavior, skill, and confidence is warranted.

## Conclusions

The findings of this review suggest SEL programs administered at a relatively low intensity may be an effective way to increase social competence, emotional competence, behavioral self-regulation, and early learning outcomes and reduce behavioral and emotional difficulties in children aged 2 to 6 years. The SEL interventions appear to be particularly successful at increasing emotional knowledge, understanding, and regulation. To better understand the active ingredients and core components of successful programs and the sustainability of program benefits over time, longitudinal research that includes comprehensive and thorough measures of social, emotional, and cognitive functioning is recommended.

## Notes

Supplement. **eFigure.** Example Search Strategy **eTable 1.** Descriptive Summary of 81 Studies Examining Universal Social and Emotional Learning Programs in Preschool Settings **eTable 2.** Social and Emotional Learning Program Descriptions **eTable 3.** Summary of Constructs Within Each Domain of Social-Emotional Development and Measures Used
