# Supplementary material for: Social and Emotional Learning Associated With Universal Curriculum-Based Interventions in Early Childhood Education and Care Centers: A Systematic Review and Meta-analysis
Source: JAMA Netw Open. 2018 Dec 7;1(8):e185727. doi: 10.1001/jamanetworkopen.2018.5727 (PMC6324369; doi:10.1001/jamanetworkopen.2018.5727)
Supplement: Supplement. — eFigure. Example Search Strategy eTable 1. Descriptive Summary of 81 Studies Examining Universal Social and Emotional Learning Programs in Preschool Settings eTable 2. Social and Emotional Learning Program Descriptions eTable 3. Summary of Constructs Within Each Domain of Social-Emotional Development and Measures Used [file jamanetwopen-1-e185727-s001.pdf]

Supplementary Online Content

Blewitt C, Fuller-Tyszkiewicz M, Nolan A, et al. Social and emotional learning associated with universal curriculum-based interventions in early childhood education and care centers: a systematic review and meta-analysis. *JAMA Netw Open*. 2018;1(8):e185727. doi:10.1001/jamanetworkopen.2018.5727

eFigure1. Example Search Strategy

eTable 1. Descriptive Summary of 81 Studies Examining Universal Social and Emotional Learning Programs in Preschool Settings

eTable 2. Social and Emotional Learning Program Descriptions

eTable 3. Summary of Constructs within each Domain of Social-Emotional Development and Measures Used

This supplementary material has been provided by the authors to give readers additional information about their work.

© 2018 Blewitt C et al. *JAMA Network Open.*

eFigure1. Example Search Strategy

Databases: PsychInfo, Medline Complete, ERIC

Limiters: Peer-reviewed, published between January 1990 and December 2017

No limits on language were applied. Studies that were not available in English were translated via Google Translate where format allowed (e.g. pdf file). Papers saved in a file format that could not be translated to English in Google Translate were excluded (e.g. pic file)

Intervention* OR program* OR curricul*

AND

“early learning centre” OR “early learning center” OR preschool* OR “pre school” OR “pre-school” OR childcare OR “child?care” OR kinder* OR “pre?kindergarten” OR “pre-K” OR “pre K” OR “day care” OR daycare OR “Head Start” OR “HeadStart”

AND

“social development” OR “emotion* development” OR “social learning” OR “emotion* learning” OR “social emotional learning” OR “social-emotional learning” OR “social and emotional learning” OR “SEL” OR “social skills” OR “emotional skills” OR “self-esteem” OR empathy OR “emotional intelligence” OR “conflict resolution” OR “problem?solving” OR resilien* OR aggress* OR anxi* OR prevent* OR externali* OR internali* OR withdraw*

© 2018 Blewitt C et al. *JAMA Network Open.*

eTable 1: Descriptive Summary of 81 Studies Examining Universal Social and Emotional Learning Programs in Preschool Settings

First Author (Year)	Program	Sample Size at Baseline[a]	Mean Age in Years (SD)	% Female	Nation/ Ethnicity	SES	Findings Related to Difference Between Intervention and Control Groups on Child Social-Emotional Outcomes at First Assessment Post Intervention
Allen (2009)[c]	Creating a New Generation of Peacemakers (Peacemakers Program)	161	IG 5.0 (0.1) CG 4.8 (0.5)	IG 47.5 CG 50.0	U.S., IG 74.3% White, CG 86.7% White	-	IG showed significant improvement in skills taught in program compared to CG.
Amesty (2009)	Second Step (Spanish Translation)	280	3.0-5.0	IG 49.0 CG 51.0	Venezuela	Low	Significant improvement in social knowledge (ability to identify feelings, describe non-verbal signals and generate solutions to social problems, coded as emotional competency) in IG compared to controls.
Anliak (2010)	I Can Problem Solve	83	5.0-6.0	51.8	Turkey	-	IG increased prosocial behaviour and decreased introverted behaviours compared to CG. The groups did not differ on aggression.
Anticich (2013)[c]	Fun Friends	488<br><br>*Study included intervention, active comparison (You Can Do It!) and control group*	5.4 (0.7)	55.5	Australia, majority White	Mid	Children in IG showed improved behaviour and emotional strengths at post-test compared to control and active control groups. Both IG and active control group displayed less behavioural inhibition than CG at post-test, the IG showed greater improvement than the active control group. The IG and active control group showed significantly greater reduction in behavioural difficulties at post compared to CG.

© 2018 Blewitt C et al. *JAMA Network Open.*

First Author (Year)	Program	Sample Size at Baseline[a]	Mean Age in Years (SD)	% Female	Nation/ Ethnicity	SES	Findings Related to Difference Between Intervention and Control Groups on Child Social-Emotional Outcomes at First Assessment Post Intervention
Aram (2008)	The Safe Kindergarten	92	IG 5.4 (0.3)<br>CG 5.5 (0.2)	IG 43.5<br>CG 47.8	Israel, White	Upper-Mid	IG showed greater improvement in communication skills (longer dialogue, emotionally and cognitively expressive, more clearly describe feelings and difficulties in a situation of being hurt) and social skills (number of conscious references to the story characters' inner worlds, number of solutions that the children generated for conflictual social situations) compared to CG. Groups did not differ on empathetic responding, information recalled or percentage of effective solutions generated in a structured assessment of social information processing.
Arda (2012) [c]	PATHS	95	6.0	IG 51.9<br>CG 48.9	Turkey	-	Children who participated in the program showed significantly greater improvement than comparison peers in aggressive/disruptive behaviours, concentration/attention and social-emotional competence, based on teacher report. No significant difference in children's receptive emotional vocabulary.
Ashdown (2011)	You Can Do It! Early Childhood Education Program	100<br><br>*Prep children only = 42*	5.0	45.5	Australia, mixed	Low	Teachers reported significantly improved social-emotional wellbeing, social-emotional competence and social skills in IG compared to CG. Intervention did not appear to decrease problem behaviours or increase reading level.
Barnett (2008)	Tools of the Mind	210	3.0-4.0	47.1	U.S., 92.6% Latino	Low	Intervention predicted significant reduction in problem behaviours (internalising and externalising). Results indicative of improved vocabulary development (PPVT-III), however effects were small and did not reach significance in multi-level models or after adjustments for multiple comparisons. No difference between intervention and control recorded for math skill, reading decoding, early literacy, expressive vocabulary.

© 2018 Blewitt C et al. *JAMA Network Open.*

First Author (Year)	Program	Sample Size at Baseline[a]	Mean Age in Years (SD)	% Female	Nation/ Ethnicity	SES	Findings Related to Difference Between Intervention and Control Groups on Child Social-Emotional Outcomes at First Assessment Post Intervention
Bassett (2008)	ABCs of Feelings	70<br><br>Study included intervention, wait list control and active control group	3.6 - 5.5	41.4	U.S., Mixed	Low	Program led to significant increase in IG children's emotional knowledge compared to CG.
Benitez (2011)	Aprender a Convivir	147	4.0	IG 50.0<br>CG 42.0	Spain	-	IG made significantly greater improvement than CG in social cooperation, social interaction, social independence, externalising, internalising, emotional reactivity, anxious-depressive, somatic complaints, shyness, attention problems and aggress behaviour. All measures completed by teacher.

© 2018 Blewitt C et al. *JAMA Network Open.*

First Author (Year)	Program	Sample Size at Baseline[a]	Mean Age in Years (SD)	% Female	Nation/ Ethnicity	SES	Findings Related to Difference Between Intervention and Control Groups on Child Social-Emotional Outcomes at First Assessment Post Intervention
Bierman (2008)	Head Start REDI (Research-based, Developmentally Informed) Program, including PATHS	356	4.6 (0.3)	54	U.S., Mixed	Low	Participating children improved in the following skill-based measures compared to controls: vocabulary, emergent literacy skills, emotional recognition, aggressive and inept responses, and competent responses. Print awareness and emotion identification reached borderline significance. There was no difference in grammatical understanding.<br><br>In terms of child behaviour, a significant intervention effect was observed for teacher-rated aggression, observed task orientation and parent-report of language/communication. A trend towards significance was found for teacher-rated social competence, parent-rated aggression, attention problems, and reading activities, and observer-rated social competence. No differences for parent-rated social competence, observer-rated aggression or teacher-rated learning engagement or attention problems.
Boyle (2008)	I Can Problem Solve	226<br><br>*Study compared one-year instruction, two-year instruction and control group*	Kindergarten and G1	IG 55.0<br>CG 58.0	U.S., 85% Hispanic	Low	IG children who participated over two years exhibited more prosocial and less relational aggressive behaviour than both CG and children who participated for one year. On a measure of overt aggression, children with two years in program improved significantly more than controls. Using a different measure (HBRS), both instruction groups showed greater improvement in prosocial behaviour than the CG, with no difference between intervention groups. There was no significant difference between groups on aggression/impulsivity and passivity on this measure.

© 2018 Blewitt C et al. *JAMA Network Open.*

First Author (Year)	Program	Sample Size at Baseline[a]	Mean Age in Years (SD)	% Female	Nation/ Ethnicity	SES	Findings Related to Difference Between Intervention and Control Groups on Child Social-Emotional Outcomes at First Assessment Post Intervention
Brigman (1999)	Ready to Learn	145	4.0-5.0	50.0	U.S., 95% Black	-	Participating children showed significant gains in social skills and attending behaviour compared to controls. IG scored significantly higher on story structure (a subtest of listening comprehension) but not on overall score of listening comprehension.
Brigman (2003)	Ready to Learn	260	5.7	50.0	U.S., predominately White	Mid	IG scored significantly higher in listening comprehension (story structure) and behaviour compared to CG.
Carpenter (2002)	Curriculum on the Management and Promotion of Appropriate Social Skills (COMPASS)	80	4.1	46.3	U.S., 91.3% Caucasian	Low	Study did not report significant improvement in aggression or social skills post intervention.
Conner (2011)	Making Choices (adapted)	67	3.0-4.0	IG 54.8 CG 41.7	U.S., 77.9% African American	Low	Significant improvement across all measures: academic competence, social competence, peer acceptance and relationships, school performance, relationship with caregivers and aggressive hostile behaviour.
Deacon (2012)[c]	Social-Emotional Competence Development Intervention	48	5.0-6.0	54.2	South Africa, 58.3% Afrikaans, 41.7% English	-	Comparison of means at post-test indicated significant improvement in social competence and decrease in internalising behaviour. No significant effect for externalising behaviours found.
Denham (1996)	Social Emotional Intervention	130	4.1 (0.4)	-	U.S., 76% ethnic minorities	Mixed	After accounting for pre-test score and confounding variables, intervention predicted increase in social competence, productiveness, peer skills and decrease in negative affect. No significant difference between IG and CG on observed measure of positive affect.

© 2018 Blewitt C et al. *JAMA Network Open.*

First Author (Year)	Program	Sample Size at Baseline[a]	Mean Age in Years (SD)	% Female	Nation/ Ethnicity	SES	Findings Related to Difference Between Intervention and Control Groups on Child Social-Emotional Outcomes at First Assessment Post Intervention
Dereli (2009)	Social Skills Training Program for Children (The Incredible Years)	55	6.0	-	Turkey	-	Post-test scores showed significant improvement for participating children in social problem-solving and understanding of feelings.
Dereli-Iman (2014)[c]	Values Education Programme	66	5.8	-	Turkey	-	Based on pre-post difference in IG (pre-post difference in CG not significant). Significant improvement in social skills, psychosocial behaviour and problem solving.
Dobrin (2013)	Preventative Program	49	6.1(0.3)	46.9	Romania	-	Based on parent and teacher assessment at post-test, IG significantly improved social and emotional competencies.
Domitrovich (2007)	Preschool PATHS	246	IG 4.2(0.5) CG 4.4(0.5)	IG 55.0 CG 48.0	U.S., Mixed	Low	At post-test, IG showed greater improvement than CG in emotion vocabulary, knowledge of emotion expression and affective perspective taking, and less anger attribution bias. Based on teacher-report, participating children scored higher on social skills (cooperation, emotional awareness, interpersonal skills), displayed less internalising, and were less likely to be withdrawn or lacking friends compared to controls. There was no difference between groups on externalising behaviour. According to parent report, IG were more socially and emotionally competent than children in control classrooms. There were no significant group differences on parents' ratings of externalising or internalising behaviour.

© 2018 Blewitt C et al. *JAMA Network Open.*

First Author (Year)	Program	Sample Size at Baseline[a]	Mean Age in Years (SD)	% Female	Nation/ Ethnicity	SES	Findings Related to Difference Between Intervention and Control Groups on Child Social-Emotional Outcomes at First Assessment Post Intervention
Dubas (1998)	Resilient Children Making Choices (Al"s Pals: Kids Making Healthy Choices)	240	4.6	47.3	U.S., 76% African American	Described as at-risk	IG made significant improvement in behaviour compared to CG.
Fishbein (2016)	PATHS	327	Kindergar ten aged	-	U.S., predominately African American	Low	Children who took part in the program made significantly greater improvement than children in the control group on measures of aggression and internalising behaviour, social competence (emotional regulation and prosocial behaviour), impulsivity and inattention, and teacher-student relationship (conflict and closeness), peer relations and academic skills.
Flook (2015)	Kindness Curriculum	68	4.7 (0.3)	50.0	U.S., Mixed	Mixed	Significant gains in emotional regulation, sharing and delay of gratification in IG compared to CG. Children in intervention group increased prosocial behaviour, however this did not remain significant after accounting for age and gender.
Garrison (2017)	Self-Compassion and Mindfulness Training	69	4.5 (0.6)	44	U.S., 88.7% Hispanic	Low	Participating children showed significantly lower levels of emotion reactivity, withdrawal, attentional problems, aggression, sleep problems and other problems than control group children. Somatic complaints were lower in IG than GC, reaching marginal significance. A trend towards improved anxiety and depression outcomes in IG identified. There was no significant difference between groups on emotional regulation (trend towards lower lability and negativity in IG compared to CG identified).

© 2018 Blewitt C et al. *JAMA Network Open.*

First Author (Year)	Program	Sample Size at Baseline[a]	Mean Age in Years (SD)	% Female	Nation/ Ethnicity	SES	Findings Related to Difference Between Intervention and Control Groups on Child Social-Emotional Outcomes at First Assessment Post Intervention
Gavazzi (2011)	Emotional State Talk Intervention	100	4.3(0.8)	50	Italy	Mid	IG children significantly increased emotion comprehension and inner state language comprehension compared to controls. No difference between groups in terms of lingual comprehension.
Giménez-Dasí (2015)	Educational intervention program to improve emotion knowledge, emotion regulation, and social competence	57	2.2	47.4	Spain., 74% Spanish	Mid	After adjusting for cognitive development, program participation predicted increased emotional comprehension. No difference between groups for emotion regulation, social competence, anxiety/withdrawal or anger/aggression.
Gunter (2012)	Strong Start Pre-K	84<br>*Study included intervention, intervention plus booster lessons and control group*	Preschool -aged	50.0	U.S., Mixed	-	Lower levels internalising behaviour in IG compared to CG (IG showed greatest reduction initially, group who received booster lessons post-intervention showed greatest reduction over time) and less conflict in teacher-child relationship. Groups did not differ on emotional regulation, teacher-child closeness or dependency.

© 2018 Blewitt C et al. *JAMA Network Open.*

First Author (Year)	Program	Sample Size at Baseline[a]	Mean Age in Years (SD)	% Female	Nation/ Ethnicity	SES	Findings Related to Difference Between Intervention and Control Groups on Child Social-Emotional Outcomes at First Assessment Post Intervention
Hall (2008)	Stop and Think	63<br><br>*Study used a sequential cohort design. The posttest for Cohort 1 (C1) and pretest for Cohort 2 (C2) were administered on the same day so C2 could be used as a non-equivalent CG for C1.*	IG 5.9 (0.5)<br>CG 6.2 (.5)	IG 65.5<br>CG 44.1	U.S., 89.7% White, CG 100% White	-	Children who participated in program showed greater improvement in social skills and academic competence, and lower levels of problem behaviours between pre and post-test. Comparison of post-test scores with pre-test scores collected at the same time for Cohort 2 indicated changes were unlikely to be caused by maturation.
Hamre (2012)[c]	My Teaching Partner (including PATHS)	980<br><br>*Teachers assigned to one of three conditions PATHS-High, PATHS-Low and control group*	PATHS-High 4.4(0.3)<br><br>PATHS-Low 4.4(0.3)<br><br>GC 4.4(0.3)	PATHS-High 53.0<br><br>PATHS-Low 50.0<br><br>CG 51.0	U.S., Mixed	Low	Children who took part in PATHS intervention showed greater improvement in social competence, after controlling for pre-test score and wide range of child, teacher and classroom covariates. The groups did not differ at post on teacher-reported measure of social problems.

© 2018 Blewitt C et al. *JAMA Network Open.*

First Author (Year)	Program	Sample Size at Baseline[a]	Mean Age in Years (SD)	% Female	Nation/ Ethnicity	SES	Findings Related to Difference Between Intervention and Control Groups on Child Social-Emotional Outcomes at First Assessment Post Intervention
Han (2005)	Pre-k RECAP (Reaching Educators, Children and Parents)	149	4.4 (0.3)	56.0	U.S., 89% African American	Low	According to teacher report, IG children made significantly greater improvement in behaviour problems (internalising and externalising), emotional reactivity, withdrawal and attention problems than the CG. Main effect of group was non-significant for anxious/depressed, somatic complaints and aggressive behaviour subscales. Teacher-reported social skills (cooperation, assertion) were also significantly improved in the intervention group compared to comparison children, however the groups did not differ on self-control. Parent report of children's total problems or social skills did not show significant improvement in IG compared to controls.
Hughes (2015)	Promoting Alternative Thinking Strategies (PATHS)	57<br><br>*Study included intervention, an adapted version of the intervention and control group*	3.0-4.0	50.8	U.K.	Low	Children who took part in the full PATHS program improved significantly in receptive emotion vocabulary compared to controls and compared to the group of children who received an adapted version of the program. There was no difference between groups on affective perspective taking. Based on teacher report, children who took part in full PATHS improved behaviour, emotional symptoms, conduct problems, hyperactivity and prosocial scores between assessments (other groups did not show improvement). There was no statistically significant difference between groups on a measure of peer problems. No significant interactions were found for the following outcomes based on parent report: emotional symptoms, conduct problems, hyperactivity, peer problems, or prosocial skills.

© 2018 Blewitt C et al. *JAMA Network Open.*

First Author (Year)	Program	Sample Size at Baseline[a]	Mean Age in Years (SD)	% Female	Nation/ Ethnicity	SES	Findings Related to Difference Between Intervention and Control Groups on Child Social-Emotional Outcomes at First Assessment Post Intervention
Izard (2004) [c]	Emotions Based Prevention Program	116	3.8	45.0	U.S., Mixed	Low	After accounting for pre-test score and other covariates (age, gender, verbal ability), the intervention led to increase in overall emotion knowledge (emotion labelling, emotion recognition, receptive emotion vocabulary reached borderline significance) and negative emotion expression. There was no difference between groups on teacher reported social and academic competence.
Izard (2008) (Study 1)	Emotions Based Prevention Program	191	3.9 (0.6)	47.6	U.S., Mixed	Low	In a model that included several covariates, IG predicted decreased aggressive behaviour, anxious/depressed behaviour, lability/negative emotionality and negative emotion expression, and independent observer record of negative behaviour and emotions. Did not find evidence for increased emotion knowledge (however did reach statistical significance for children who were at least 4 years at pre-test), or positive or adaptive behaviour.
Jack (2009)	Second Step	102	5.5 (0.5)	56	U.S., Mixed	Majority did not receive free lunch	IG significantly decreased perpetrator behaviours compared to CG. These are indicators of aggressive behaviour in school environments.
Jakob (2005)	Second Step	56	5.0	44.6	U.S., all Caucasian	-	Children who participated in the intervention showed greater improvement at post-test in prosocial behaviour, greater decrease in aggressive behaviour and problem behaviours, and better impulse control compared to CG. There was no statistically significant difference between IG and CG on measures of attention or teacher-reported prosocial behaviour based on the Teacher Behaviour Rating Form.

© 2018 Blewitt C et al. *JAMA Network Open.*

First Author (Year)	Program	Sample Size at Baseline[a]	Mean Age in Years (SD)	% Female	Nation/ Ethnicity	SES	Findings Related to Difference Between Intervention and Control Groups on Child Social-Emotional Outcomes at First Assessment Post Intervention
Justicia-Arráez (2015)	Aprender a Convivir	313	3.0	51.7	Spain	Mid	Significant gains in IG children's social competence (cooperation, interaction and social independence) and conduct problems (internalising and externalising) compared to CG peers, based on teacher report.
King (2001)	Stop and Think (adapted)	112	6.2	50.9	U.S., 78% Caucasian	Mixed	IG demonstrated significantly lower problem and hyperactive behaviours than CG. No significant difference between IG and CG on social skills (assertion, cooperation, self-control) or learning behaviours.
Koglin (2011)	Behavioural Training for Preschool	90	5.4	47.4	Germany, Mixed	Mixed	Children who participated in the intervention showed less hyperactivity, less problems with peers, improved emotional regulation and academic skills compared to controls. No effects identified for aggressive behaviour or emotional problems. Both groups saw increase in prosocial behaviour, with no statistically significant difference between the two groups.

© 2018 Blewitt C et al. *JAMA Network Open.*

First Author (Year)	Program	Sample Size at Baseline[a]	Mean Age in Years (SD)	% Female	Nation/ Ethnicity	SES	Findings Related to Difference Between Intervention and Control Groups on Child Social-Emotional Outcomes at First Assessment Post Intervention
Landry (2014)	Responsive Early Childhood Curriculum plus explicit social-emotional classroom activities	542<br><br>*Study included RECC, RECC plus explicit social-emotional classroom activities and control group*	2.9 (0.6)	51.0	U.S., 78% African American	Low	Average of the two IGs significantly higher than CG for expressive, receptive and situation emotions understanding, with no difference between the intervention groups. No group differences found for vocabulary, early literacy, complex language or math knowledge. Average closeness in the teacher-child relationship was greater in intervention groups than control group, and average conflict was lower in intervention groups compared to controls. There was greater decline in anxiety in the RECC plus explicit SEL compared to other groups. Intervention groups showed more change over time in social competence, the group without explicit SEL showed significant difference between pre and post test, for the group with explicit SEL, a significant difference was only observed between pre and mid assessment points. No differences between group on measures of anxiety and withdrawal.
Larmar (2006)	The Early Impact Program	455	IG 4.4(0.5) CG 4.3 (0.5)	IG 50.5 CG 38.5	Australia	-	Based on teacher report, IG significantly outperformed CG at post-test on conduct problems, hyperactivity, peer relationships and prosocial behaviours, there were no differences between groups at pre-test. The groups did not significantly differ on anxiety at post-test (though did at follow up). Parent report did not identify significant differences between groups at post-test for conduct problems, hyperactivity, emotion problems, peer problems or prosocial behaviour.
Lewis (2012)	Fun FRIENDS	110	5.11	-	U.S.	-	Anxiety symptoms increased from pre to post for children in the intervention school whereas they decreased for children in the control school.

© 2018 Blewitt C et al. *JAMA Network Open.*

First Author (Year)	Program	Sample Size at Baseline[a]	Mean Age in Years (SD)	% Female	Nation/ Ethnicity	SES	Findings Related to Difference Between Intervention and Control Groups on Child Social-Emotional Outcomes at First Assessment Post Intervention
Lonigan (2015)	PATHS	855<br><br>*Participants assigned to one of three conditions. Explicit: PATHS and classroom and behaviour management skills, Implicit: classroom behaviour and management skills only and control*	4.5 (0.4)	53.0	U.S., Mixed	Mixed	Positive impacts for both intervention groups on language, phonological awareness, math, and socioemotional outcomes, but there were no added benefits to academic or socioemotional outcomes (including anger and aggression and anxiety) for the children receiving explicit SEL instruction.

© 2018 Blewitt C et al. *JAMA Network Open.*

First Author (Year)	Program	Sample Size at Baseline[a]	Mean Age in Years (SD)	% Female	Nation/ Ethnicity	SES	Findings Related to Difference Between Intervention and Control Groups on Child Social-Emotional Outcomes at First Assessment Post Intervention
Lösel (2006)	Parent and Child Training Program Package, based on I Can Problem Solve	168<br><br>*Study evaluated the effectiveness of a child social skills training program, parenting training, and a combination of both. Data reported relates to kindergarten intervention.*	4.0-5.0	55.2	Germany	Mixed	Significant improvement in total problems and emotional problems for IG compared to CG. No significant difference between groups on measure of social problems or hyperactivity/inattention.
Lynch (2004) (1996-97 Michigan Controlled Study)	Al's Pals: Kids Making Healthy Choices	399	IG 4.4<br>CG 4.3	IG 50.0<br>CG 48.5	U.S., Mixed	Low	Significant improvement in IG on the Child Behaviour Rating Scale, and social independence and problem behaviour subscales of the Child Behaviour Rating Scale, compared to CG.
McKinney (1998)	Taking Part	29	4.1 (0.6)	58.6	U.S., 100% African American	-	Significant difference between IG and CG on teacher-rated problem behaviour. No difference for parent rated social skills, problem behaviours or teacher rated social skills.
Mishara (2006)	Zippy's Friends	418<br><br>*Kindergarten classes only*	IG 6.2 (0.4)<br>CG 6.1 (0.4)	46.7	Canada<br><br>Research conducted in Lithuanian kindergartens	-	Greater improvement in IG compared to CG on measures of assertion, self-control, cooperation and empathy, externalising, hyperactivity and coping strategies. No differences between groups on internalising.

© 2018 Blewitt C et al. *JAMA Network Open.*

First Author (Year)	Program	Sample Size at Baseline[a]	Mean Age in Years (SD)	% Female	Nation/ Ethnicity	SES	Findings Related to Difference Between Intervention and Control Groups on Child Social-Emotional Outcomes at First Assessment Post Intervention
Moisan (2014)	Promoting Social Competence in Classrooms	182<br><br>*Universal program, assessment focused on children with elevated aggression. Study included PSC, PSC plus dyadic peer intervention and control group*	Kindergarten	31.0	Canada, 97% Canadian	Mid	Children in experimental conditions showed better social problem-solving skills compared to children in the control group. A marginal increase also reported for cooperation in the two experimental conditions. Overall, the impact of the PSC DPI condition was not superior to the PSC only condition.

© 2018 Blewitt C et al. *JAMA Network Open.*

First Author (Year)	Program	Sample Size at Baseline[a]	Mean Age in Years (SD)	% Female	Nation/ Ethnicity	SES	Findings Related to Difference Between Intervention and Control Groups on Child Social-Emotional Outcomes at First Assessment Post Intervention
Morris (2014)[c, e]	Head Start CARES Demonstration Project (including PATHS, Tools of the Mind-Play and Incredible Years Teacher Training. Incredible Years Teacher Training not considered curriculum-based SEL in this review)	2,114	4.4	48.8	U.S., Mixed	Low	*PATHS:* Children in IG were better able to identify emotional expressions of faces, emotions in pictures and in short scenarios, and were better able to generate competent responses to peer-provocation scenarios compared to CG peers. Teachers reported increased positive social behaviours in IG compared to controls. Based on teachers' reports and interviewers' assessments, no difference between groups on behaviour problems and executive function skills, with exception of teacher's report of children's learning behaviours which was greater in IG compared to CG.<br><br>*TOOLS:* No difference between groups on behaviour regulation, executive functioning skills or learning behaviours. IG children were better able to identify emotions correctly compared to CG. Children did not however show more competent social problem-solving solutions, or higher social behaviours based on teacher report.
O'Connor (2014)	INSIGHTS	435<br><br>*Intervention delivered in Kindergarten and G1*	5.4 (0.6)	48	U.S., 75% Black, non Hispanic	Low	IG displayed greater growth in math and reading ability, improved attention and greater decreases in behaviour problems over time, compared to CG children.

© 2018 Blewitt C et al. *JAMA Network Open.*

First Author (Year)	Program	Sample Size at Baseline[a]	Mean Age in Years (SD)	% Female	Nation/ Ethnicity	SES	Findings Related to Difference Between Intervention and Control Groups on Child Social-Emotional Outcomes at First Assessment Post Intervention
Opre (2013) [c]	Rational Emotive Education Program Social Emotional Learning Facilitator Kit (SELF Kit)	223<br><br>*Study (#1) included intervention, active control and control groups, facilitated by teachers with and without REE expertise*	4.0-6.0	52.0	Romania	-	Children in IG with a teacher with REE expertise demonstrated greater improvement in social, emotional and behavioural skills compared to non-systematic REE intervention groups, regardless of expertise.
Ornaghi (2017)	Conversational Approach	95	2.5 (0.3)	43.2	Italy	Mid	After controlling for age and general language ability, IG children performed better on measures of emotion knowledge and emotional state talk, and demonstrated more prosocial behaviour than CG peers. The intervention did not have a significant effect on levels of aggression.
Ornaghi (2015)	Conversational Approach	75	5.1 (0.6)	45.3	Italy	Mid	Greater improvement in objective assessment of prosocial orientation and emotion comprehension in IG compared to comparison children. There was no difference between groups on false belief understanding.
Ostrov (2015)	Early Childhood Friendship Project - Revised	141	3.8 (0.6)	47.5	U.S., Mixed	Mid	Intervention reduced relational bullying in the IG compared to CG, and reduced relational and physical victimisation for girls in the IG relative to the CG.

© 2018 Blewitt C et al. *JAMA Network Open.*

First Author (Year)	Program	Sample Size at Baseline[a]	Mean Age in Years (SD)	% Female	Nation/ Ethnicity	SES	Findings Related to Difference Between Intervention and Control Groups on Child Social-Emotional Outcomes at First Assessment Post Intervention
Pahl (2010)	The Fun FRIENDS Program	263	4.6	47.9	Australia	Mixed	Teacher report indicated lower behavioural inhibition and higher social competence in IG compared to CG at post-test. No significant differences in behaviour inhibition, social competence or anxiety were identified via parent report at post-test
Peterman (2008)	Project Primer	95<br>*Children in preschool classes only*	5.0	49.2	Germany	Mixed	IG children displayed lower burden of social-emotional problems compared to control group children. The intervention did not lead to significant differences between IG and CG on measures of social competence, emotional competence and aggressive behaviour.
Pickens (2009)[c]	The Peace Education Foundation Socio-Emotional Development Program	296	4.0 (0.7)	51.0	U.S., Mixed	-	Based on teacher report, participating children showed greater social cooperation, social interaction, social independence, and lower levels of externalising and internalising behaviours compared to controls.
Poehlmann-Tynan (2016)	Kindness Curriculum (Adapted)	29	3.9 (0.5)	49.0	U.S., 72% Non-White	Low	Participating children significantly improved integrated self-regulation compared to controls. Significant differences not reported for empathetic responding, representations of empathy, or representations of compassion.
Randall (2011)	First Friends	87	5.4	41.4	Canada	Low	IG demonstrated significantly stronger observed socio-emotional and social cognitive abilities, more prosocial behaviours, and less negative behaviours compared to a control group. Parent and teacher reports did not reveal significant changes

© 2018 Blewitt C et al. *JAMA Network Open.*

First Author (Year)	Program	Sample Size at Baseline[a]	Mean Age in Years (SD)	% Female	Nation/ Ethnicity	SES	Findings Related to Difference Between Intervention and Control Groups on Child Social-Emotional Outcomes at First Assessment Post Intervention
Reid (2007)	The Incredible Years Child Training Curricula	340<br><br>*Delivered as a universal program see Webster-Stratton et al., 2008. This paper focused on children with moderate – high risk behaviour. Study included intervention, intervention and home component and control groups*	5.6	40.9	U.S., Mixed	Low	Significant difference between IG and CG on teacher reported externalising measured by Social Competence and Behaviour Evaluation – Preschool Edition. No differences between children who took part in classroom based version of program and CG on measures of negative behaviour, externalising and internalising based on CBCL, prosocial behaviour, emotional regulation or social competency.
Rodker (2013)	Zippy's Friend	127	5.0	41.6	U.S., Mixed	-	Teachers reported greater social skills in IG compared to CG. No differences recorded for parent-rated social skills, parent or teacher rated problem behaviours, theory of mind or affect recognition
Saltali (2010)	Emotional Education Program, based on PATHS	64	6.0	-	Turkey	-	In a post-test comparison, participating children demonstrated greater emotion understanding, emotion identification and emotion expression compared to control group children.

© 2018 Blewitt C et al. *JAMA Network Open.*

First Author (Year)	Program	Sample Size at Baseline[a]	Mean Age in Years (SD)	% Female	Nation/ Ethnicity	SES	Findings Related to Difference Between Intervention and Control Groups on Child Social-Emotional Outcomes at First Assessment Post Intervention
Sandy (2000)	Peaceful Kids Conflict Resolution Program	248<br><br>*Participants assigned to one of three conditions: staff and child training; staff, child and parent training; and CG. Sample size is based on number of participants included in analyses*	2.5-6.0	54.0	U.S., Mixed	Low	Children who took part in staff, child and parent training showed greater improvement in response to conflict situations, improved assertiveness, self-control and reduced internalising compared to children in control or staff and child training conditions. Children in staff, child and parent training showed greater cooperation than children in staff and child training, and reduced internalising compared to staff and child training.
Schell (2015) [b]	Lubo from Outer Space	221	IG 5.2 (0.5)<br>CG 5.2 (0.4)	IG 54.8<br>CG 49.5	Germany	Mixed	Greater improvement in social problem solving in IG compared to control, in a mixed model adjusting for confounding variables (gender, age, disease/ disability, baseline). Group differences on measures of externalising, problem behaviours, internalising, prosocial behaviour were not significant after adjusting for confounding variables.
Schmitt (2014) [c]	The Positive Action Program	135	Preschool -aged	46.0	U.S.	-	Compared to CG, IG children displayed significantly improved outcomes across the total scale score and all subscale scores in in assessment of the 11 different domains addressed by the program.

© 2018 Blewitt C et al. *JAMA Network Open.*

First Author (Year)	Program	Sample Size at Baseline[a]	Mean Age in Years (SD)	% Female	Nation/ Ethnicity	SES	Findings Related to Difference Between Intervention and Control Groups on Child Social-Emotional Outcomes at First Assessment Post Intervention
Schmitt (2017)	The Positive Action Program	75	4.3 (0.6)	47.0	U.S., Mixed	Low	Findings showed positive direction on children's social problem solving and parent-rated social-emotional competence although this did not reach statistical significance. Teachers reported IG children decreased in social-emotional skills. There was no difference between groups on aggressive problem solving.
Serna (2000) [b]	Self-Determination Program	84	4.5	44.0	U.S., 71.4% Hispanic	Low	Children who took part in the program demonstrated significantly greater improvements in problem behaviour, inattention/overactivity, adaptive behaviour and social interaction than comparison children based on teacher report. No difference observed on maladaptive behaviour, aggression, oppositional/defiant, overall functioning and critical events. Parent report showed statistically significant improvement in social skills for intervention group children compared to controls, with no differences between groups on problem behaviours or communication.
Serna (2003)[b]	Self-Determination Program	111	4.1	50.0	U.S., 82% Hispanic	Low	IG showed greater change in problem behaviours and ADHD symptoms compared to CG. No differences between groups for adaptive behaviour, social interaction, maladaptive behaviour, aggression, or critical events.
Seyhan (2017) [c]	PATHS Preschool Program	565	4.0-6.0 years	47.0	Turkey	Mid	No significant differences between groups were found at pre-test. At post-test, children who participated in the program showed greater social-emotional skills, interpersonal relationship skills and emotional regulation, more positive teacher-student relationship and greater dependency in the teacher-student relationship. There were no differences in the levels of conflict and closeness in the student-teacher relationship based on teacher report.

© 2018 Blewitt C et al. *JAMA Network Open.*

First Author (Year)	Program	Sample Size at Baseline[a]	Mean Age in Years (SD)	% Female	Nation/ Ethnicity	SES	Findings Related to Difference Between Intervention and Control Groups on Child Social-Emotional Outcomes at First Assessment Post Intervention
Starnes (2017)	Second Step	63	5.2	57.1	U.S.	Upper-mid	At post-test, children in IG exhibited greater verbal and quantitative reasoning and mathematics than CG. No difference for early literacy achievement.
Stefan (2013)	Social Emotional Prevention Program	158 (note: sample at baseline = 204, however demographics reported for final sample of 158)	IG 4.2<br>CG 4.1	IG 52.8<br>CG 58.0	Romania	Mid – High	After accounting for age, gender and pre-test score, intervention status predicted improved ability to name emotions, recognise emotions, positive solutions for possible conflict situations, teacher-rated social competence, emotional competence and reduced externalising problems. No effect identified for teacher-rated internalising.
Stephenson (2009)	Second Step	41	3.9 (0.5)	41.5	U.S., Mixed	Mixed	No differences between IG and CG found for anti-social behaviour, overt/physical aggression, or relational aggression.
Tominey (2011)	Red Light Purple Light	65	4.6	60.0	U.S.	Mixed	No treatment effect found for behavioural self-regulation after controlling for covariates (however improvement in the expected direction). Post hoc tests showed participation in IG was related to self-regulation gains in children who started the program with low skill level. IG children showed significantly greater improvement in letter-word identification compared to CG children. Scores in applied problems and picture vocabulary did not differ between groups.
Ulutaş (2007)	Emotional Intelligence Education	120	6.0	50.0	Turkey	NR	IG children demonstrated improved emotional intelligence and empathy at post-test, compared to CG, after accounting for pre-test scores.

© 2018 Blewitt C et al. *JAMA Network Open.*

First Author (Year)	Program	Sample Size at Baseline[a]	Mean Age in Years (SD)	% Female	Nation/ Ethnicity	SES	Findings Related to Difference Between Intervention and Control Groups on Child Social-Emotional Outcomes at First Assessment Post Intervention
Upshur (2017)	Second Step Early Learning (SSEL) Curriculum – expansion of Second Step (SEL + EF)	492	4.5 (0.3)	49.8	U.S., Mixed	Low	Controlling for baseline executive functioning, social-emotional skills, cognitive ability, parent income, child gender, age, and ethnicity, participating children had marginally better end of preschool social-emotional skills.
Upshur (2013)	Second Step Pre/Kindergarten Social and Emotional Learning	Year 1 233<br>Year 2 177	Year 1<br>IG 3.9 (0.7)<br>CG 4.2 (0.7)<br><br>Year 2<br>IG 3.7 (0.7)<br>CG 4.0 (0.7)	Year 1<br>IG 40.9<br>CG 54.0<br><br>Year 2<br>IG 35.1<br>CG 50.8	U.S., Mixed	Mixed	In Year 1, the IG group showed greater decrease in behaviour problems than CG, however this was not significant after accounting for baseline score. No differences between IG and CG on prosocial skills in year 1, or on prosocial skills or behaviour problems in year 2.
Vestal (2001)	40-hour College-level course, including I Can Problem Solve curriculum.	64	IG 4.9 (0.6)<br><br>CG 4.9 (0.5)	IG 53.6<br><br>CG 46.4	U.S., IG 73.0% Black,<br>CG 74.1% Black	Low	Children in IG provided more relevant solutions, higher relevancy scores and lower force ratio scores than children in the comparison group.

© 2018 Blewitt C et al. *JAMA Network Open.*

First Author (Year)	Program	Sample Size at Baseline[a]	Mean Age in Years (SD)	% Female	Nation/ Ethnicity	SES	Findings Related to Difference Between Intervention and Control Groups on Child Social-Emotional Outcomes at First Assessment Post Intervention
Webster-Stratton (2008)[d, e]	Incredible Years (IY) Teacher Classroom Management and Child Social and Emotion curriculum (Dinosaur School)	1,768	5.3 (1.1)	50	U.S., Mixed	Low	Observations showed significant improvement in children's emotional self-regulation, social competence and conduct problems compared with the control children. Effect sizes were strongest for children from classrooms with the poorest initial scores, and significant main were effects identified for conduct problems, disengagement, time in solidary play, positive interactions with teacher, positive interactions with peers and time spent engaging with peers. Conduct problems and disengagement significantly differentially improved in the intervention classrooms compared to the control condition. Children from classrooms that were most at initial risk benefited most from the intervention.

[a] Where studies included preschool and primary school aged children and data for preschool and primary school aged children was reported separately, sample size is based on preschool-aged children.
[b] S =Social; E=Emotional; P = Problem Behaviour and Emotions; B= Behavioural Self-Regulation; L = Early Learning Outcomes
[c] Study not included in meta-analysis
[d] Certain data included in meta-analysis
[e] Due to time constraints, authors not contacted for additional data

© 2018 Blewitt C et al. *JAMA Network Open.*

eTable 2: Social and Emotional Learning Program Descriptions

Intervention	Program Aim	Skills Targeted/Program Approach	Person Delivering Program	Intensity and Program Length	Parental Involvement	Relevant Studies
ABCs of Feelings	To increase emotional knowledge	Focus on emotional knowledge. Each concept is taught and reinforced before the next is introduced. The program alternates between one week of learning a new concept and one week of reinforcement and integration.	Facilitator, California School of Professional Psychology Trainees selected by their supervisor to participate in the study	Once a week, 10-20 minutes for 12 weeks, delivered to small group (5-8 children)	Weekly newsletter	Bassett (2008)
Aprender a Convivir	To develop social competence and deter development of risk behaviours.	Program divided into four blocks sequenced according to the participants' developmental process; Block 1: rules and rule-following, Block 2: feelings and emotions, Block 3: communication skills and Block 4: help and cooperation. Each session starts with a group activity followed by both individual activities (colouring handouts, working with play dough, puzzles) and group activities (games, songs, role-playing, tales) allowing the children to verbalise what they are doing and why.	Teacher	1.5 hours per week (2 x 45 minute sessions), 4 blocks taking 3 weeks each	First session of each block included planned activity with parents, aimed to create dialogue between parents and children, and reinforce and encourage the transfer of classroom content.	Benitez (2011)
			Teacher and specialist	2 times per week for 12 weeks	-	Justicia-Arráez (2015)
Al's Pals: Kids Making Healthy Choices.	To identify and understand feelings, express and respond in prosocial ways, appreciate different ideas, think flexibly, distinguish between safe and	During each lesson, teacher introduces key concepts that are reinforced throughout the week. Lessons included games, guided creative play, extensive use of puppetry, children's books,	Teacher	43 lessons, 20 minutes per lesson for 6 months	-	Dubas (1998)

© 2018 Blewitt C et al. *JAMA Network Open.*

Intervention	Program Aim	Skills Targeted/Program Approach	Person Delivering Program	Intensity and Program Length	Parental Involvement	Relevant Studies
	unsafe substances and situations, make healthy choices and solve interpersonal conflict in non-violent ways.	photographs, and original songs to convey health-promoting concepts and to enhance prosocial life skills.	Teacher	2 times per week (15-20 minutes per lesson) for 23 weeks	Offers methods to communicate to parents. Parent letters explain what is being taught in the curriculum and offer activities for home.	Lynch (2004)
Behavioural Training for Preschool	To reduce aggressive, shy and withdrawn behaviour.	Skills are promoted in three domains: emotional competencies, problem-solving skills and social skills.<br>Program is based on a story about two mermaids, a boy and a girl of preschool-age and a dolphin. Stories used to motivate children and to introduce typical social problems. Discussions, role-play and games included. Units focus on recognising and naming feelings and solving age-appropriate conflicts. Lesson sequence is based on the social information processing model.	Teacher	1-2 lessons per week for an average 35 minutes.	-	Koglin (2011)
Conversational Approach	To train preschool children in the comprehension of the nature, causes, and regulation of emotions.	Sessions held during circle time. Three training sessions devoted to each of the four target emotions: during the first session, children were trained in understanding the nature of the target emotion, in the second lesson, children focused on understanding its causes, and in the third session, on understanding that it is possible to regulate this emotion. Children encouraged to converse with their peers about the nature, causes, and regulation of emotions, drawing on their own personal experience.	Researcher	2 times per week for 6 weeks. Each session lasted around 1 hour. Delivered to small group (5-6 children)	-	Ornaghi (2015)
			Teacher	15 minutes daily for 2 months. Delivered to small group (4-5 children)	-	Ornagi (2017)

© 2018 Blewitt C et al. *JAMA Network Open.*

Intervention	Program Aim	Skills Targeted/Program Approach	Person Delivering Program	Intensity and Program Length	Parental Involvement	Relevant Studies
Curriculum on the Management and Promotion of Appropriate Social Skills (COMPASS)	To teach basic individual social skills.	Program includes instructional and rehearsal components. During instruction, teachers model inappropriate and appropriate behaviours using hand puppets. Social skills included cooperation, participation, validation/support, and communication. To rehearse, children role-play appropriate behaviours with hand puppets with a play partner during a cooperative activity.	Teacher	2 times per week, 15 minutes circle time and small group practice for 6 weeks	-	Carpenter (2002)
Early Childhood Friendship Project – Revised	To reduce physical and relational aggression, peer victimisation, and increase prosocial behaviours.	Updated program includes additional lessons focusing on helping and sharing behaviour, and verbal aggression. Each week involves 4 intervention blocks: a lesson facilitated by puppets, in vivo practice through reinforcement during free play, a passive participatory activity (e.g., craft), and an active participatory activity (e.g., game).	PhD students in child clinical psychology or early childhood education	8 weeks	-	Ostrov (2015)
Educational intervention program to improve emotion knowledge, emotion regulation, and social competence	To improve emotion knowledge and regulation, and social competence.	Methods include fiction, play, and storytelling. Three marionette characters are used to present the activities to children and provide a connecting theme between the sessions. Activities aimed at the three basic components (identification, causality, and labelling) of the four basic emotions (happiness, sadness, anger and fear). For negative emotions (sadness, anger and fear), the program also introduces basic emotion regulation strategies. Components and emotions were introduced according to the developmental pattern of acquisition.	Teacher	Weekly 30 minute sessions for 6 months	-	Giménez-Dasí (2015)

© 2018 Blewitt C et al. *JAMA Network Open.*

Intervention	Program Aim	Skills Targeted/Program Approach	Person Delivering Program	Intensity and Program Length	Parental Involvement	Relevant Studies
Emotion State Talk Intervention	To train preschool children to use emotional state talk.	Children were read illustrated stories enriched with emotional state talk. At the end of each story, children took part in games designed to elicit emotional mental state talk.	Researcher	2 times per week, storybook and 12-15 minutes discussion, for 2 months. Delivered to small groups (6-7 children)	-	Gavazzi (2011)
Emotional Intelligence Education	-	-	-	Over 2 days each week for 12 weeks	-	Ulutaş (2007)
Emotions Based Prevention Program	To increase children's ability to understand and regulate emotions, utilise modulated emotions and reduce maladaptive behaviour.	Program relies on the intrinsic benefits that derive from increased emotional competence and decreased maladaptive behaviour. Part 1 of each lesson begins with the teacher leading a puppet show to illustrate the concepts in the lesson. The rest of the lesson includes interactive games relating to aspects of emotions. The teacher asks the children if they would like to tell the class what causes them to feel the emotion featured in the lesson (e.g., "What makes you feel sad?"). Each lesson ends with the interactive reading of an emotion storybook that provides children the opportunity to experience mild emotions vicariously.	Teacher	20 lessons over approx. 20 weeks	-	Izard (2004)
			Teacher	20 lessons over 20 weeks	Parents sent a weekly message that summarised the lesson of the week or highlighted a key aspect of it. It also requested that the parent complete and return a brief parent–child lesson-related activity that requires a response from the child. Four meetings held for parents (poor attendance noted)	Izard (2008)
First Friends	To promote the following social skills: problem solving, conflict resolution, planning, emotional understanding, empathy, assertiveness, anger	Each session includes teaching a specific social skill, a play session where children are encouraged and motivated to practice the skills learned, and individual strategies during play where group facilitators chose	Two facilitators	Weekly 30 minute sessions for 8 weeks, groups	-	Randall (2003)

© 2018 Blewitt C et al. *JAMA Network Open.*

Intervention	Program Aim	Skills Targeted/Program Approach	Person Delivering Program	Intensity and Program Length	Parental Involvement	Relevant Studies
	management, verbal communication, creativity, and cooperation.	specific interventions based on the individual child's needs and abilities.		of 6 to 8 children		
Fun Friends	To reduce anxiety and behavioural inhibition, and enhance social and emotional competence in young children.	Cognitive-behavioural, using relaxation, cognitive restructuring, attention training and graded exposure to anxiety provoking situations and problem solving.<br><br>Fun FRIENDS is an acronym for the strategies taught in the program: Feelings, Remember to Relax, I Can Do My Best, Encourage, Nurture, and Don't Forget to be Brave. Program includes relaxation, cognitive restructuring, attention exercises and gradual exposure to and problem solving within situations that provoke anxiety.	Teacher	Once weekly across 2 terms (12 sessions)	Family learning adventure workbook provided step-by-step instruction for home implementation of session skills. Two parent meetings held during the implementation period. Content includes child anxiety, social and emotional competence and resilience.	Anticich (2013)
			Post-graduate psychology student	1 hour per week for 9 weeks	Parents invited to attend three parent information sessions on anxiety psychoeducation and information regarding session content. Parents provided with weekly handouts outlining session content and suggestions for home reinforcement of skills.	Pahl (2010)
			School guidance counsellor	One session per week for 15 sessions, approximately 35-45 minutes per session,	One hour information session and two hour-long parent groups across the intervention, poorly attended.	Lewis (2012)

© 2018 Blewitt C et al. *JAMA Network Open.*

Intervention	Program Aim	Skills Targeted/Program Approach	Person Delivering Program	Intensity and Program Length	Parental Involvement	Relevant Studies
				including small group activities. Booster 4 weeks after program ended.		
I Can Problem Solve (ICPS)	To teach children how to solve problems and prevent possible problem situations<br><br>Inter-personal cognitive problem-solving skills	Lessons focus on training children to generate a variety of solutions to interpersonal problems, think about the consequences of each potential solution and identify thoughts, feelings, and motives that can generate problem situations. The child, rather than the teacher, must solve the problem. Program includes formal lessons and specific suggestions for integrating learning into the day-to-day classroom.	Teacher	83 lessons over approx. 4 months, 20-30 minutes per lesson	Monthly letters sent to parents regarding the intervention including activities that could be used at home in problem situations	Anliak (2010)
			Teacher	2 lessons per week for 4 months	-	Boyle (2009)
			Translated as “trained trainer”	15 sessions, 30-60 minutes per session, 3-5 sessions per week, delivered to small groups	-	Lösel (2006)
INSIGHTS	To support children’s ability to self-regulate by enhancing their attentional and behavioural repertoire.	During the first 4 weeks, children are introduced to four puppets with different temperaments. Children explore how, on the basis of a puppet’s temperament, some situations are easy and others are challenging. Children then work with the puppets to apply problem-solving strategies when confronted with daily problems.	Facilitator with teachers engaged	Over 10 weeks, the classroom program was delivered in 45-minute lessons	Teachers and parents attended 10 weekly 2-hr facilitated sessions based on a structured curriculum. Parents were given assignments to complete between sessions.	O’Connor (2014)

© 2018 Blewitt C et al. *JAMA Network Open.*

Intervention	Program Aim	Skills Targeted/Program Approach	Person Delivering Program	Intensity and Program Length	Parental Involvement	Relevant Studies
Intensive Teacher Training (based on I Can Problem Solve)	To instruct teachers in the theory of conflict, conflict management, socio-emotional development and a problem-solving curriculum for preschool students.	Over seven weeks, teachers took part in a 13-session college-level course. Topics covered instruction and theories on conflict, peace education, conflict resolution, and emotional and social development. Teacher's took part in lectures, role plays, discussions, and presentations. Teachers were trained in the I Can Problem Solve curriculum.	Teacher	Curriculum implemented for 2 months	-	Vestal (2001)
Kindness Curriculum	Attention and emotional regulation training to cultivate kindness and care towards oneself and others.	Composed of eight themes (mindful bodies and planting seeds, I feel emotions on the inside, how I feel shows on the outside, taking care of strong emotions, calming and working out problems, gratitude, all people depend on each other and the earth, and gratitude and caring for our world). Each session includes breathing and movement exercises, music, reading children's literature about kindness and caring, and activities that provide opportunities to increase awareness of inner and outer experiences of emotions, sharing, and kind acts.	Experienced mindfulness instructors	20–30 minute lessons twice per week for 12 weeks	-	Flook (2015)
			Experienced mindfulness instructors	20–30 minute lessons twice per week for 12 weeks	-	Poehlmann-Tynan (2016)
Lubo from Outer Space	To promote knowledge, expression, and regulation of emotions, teach social conflict and problem-solving strategies, and promote children's abilities of perspective taking and building friendships.	Program embedded in a story about "extra-terrestrial Lubo" (hand puppet), who travels to Earth to learn about feelings and friendship. "Lubo" keeps running into social problems, which children help to solve. Methods include cooperative games, role-play, discussions, picture cards, creative methods, and a feedback system to support the learning process in a stimulating way.	Teacher under guidance of Lubo worker	3 times per week, 35-40 minutes per lesson for 12 weeks. Delivered to groups of 9-14 children	Parents received monthly letters explaining the key concepts to be taught in the upcoming month and suggestions on how to incorporate these in daily practice at home	Schell (2015)

© 2018 Blewitt C et al. *JAMA Network Open.*

Intervention	Program Aim	Skills Targeted/Program Approach	Person Delivering Program	Intensity and Program Length	Parental Involvement	Relevant Studies
Making Choices	To reduce aggressive and hostile behaviour by strengthening SIP skills, developing emotional-regulation skills (e.g., self-talk and impulse control), providing structured opportunities for pro-social peer involvement and increased peer acceptance.	Activities structured to correspond to the steps in SIP. Divided into units, lessons had specific skill-related goals and activities.	Social work student and a master's-level teacher co-facilitated	2 x 20-minute sessions per week for 14 weeks, delivered to small groups of 4 children	Included family education intervention, *Strong Families*	Conner (2011)
Peace Education Foundation Socio-Emotional Development Program	The curriculum addresses conflict resolution, anger management and communication skills to promote children's positive interactions, cooperation and emotional self-regulation.	Program instructs teachers and parents how to use activities and ''I-Care Rules and Language'' to encourage empathy and fair play, express feelings, avoid conflict, manage anger and interact more positively with others.	Teacher	1 year	Parents participated in *Creating Caring Children* and *Peace-making Skills for Little Kids* training (3 hours)	Pickens (2009)
Peaceful Kids Conflict Resolution Program	To promote social-emotional, cognitive and conflict resolution skills.	Circle time sessions relying primarily on hands-on group activities (e.g. modelling by adults, puppetry, stories, role plays), allowing children to analyse situations and behaviours, identify possible causes and consequences of characters actions, and apply problem solving skills.	Peaceful Kids Faciliator	1 session per week for 20-40 minutes, for 15 weeks	Program for parents included four 2-hr workshops (focused on parenting skills through adult-to-adult practice) and were provided take home activities twice weekly to complement work in the classroom.	Sandy (2000)
Pre-K RECAP	To learn a common language and set of skills for functioning adaptively, developing pro-social classroom norms and expectations for children's interactions with each other.	Incorporates some materials (e.g., puppets, pictures) from Second Step. Lessons reinforced daily by teachers using positive tokens, teacher modelling and mediation of problem-solving steps, and explicit discussion of behavioural and affective consequences of behaviour choices.	Teacher	2-3 times per week for 9 months, reinforced daily.	Included parent program to enhance parent skill in supporting children's prosocial behaviour, parent attendance was very low (<5%).	Han (2005)

© 2018 Blewitt C et al. *JAMA Network Open.*

Intervention	Program Aim	Skills Targeted/Program Approach	Person Delivering Program	Intensity and Program Length	Parental Involvement	Relevant Studies
Preventative Program	To enhance social and emotional competencies.	Focus on emotional literacy, labelling of emotional states, identification of emotional markers, empathy, emotion-regulation strategies, anger and impulsivity control, problem-solving abilities and social competencies. Methods include direct teaching, stories, role plays, puppet shows and situational scaffolding.	-	3 times per week (20-30 minutes each) for 5 months	-	Dobrin (2013)
Project Prim!r	To improve children's emotional knowledge, understanding and regulation, social information processing, prosocial and problem-solving skills, and to prevent oppositional defiant and aggressive behaviour.	Based on cognitive-behavioural methods and tasks including contingency programs and role play.	Teacher	1-2 sessions per week (25 sessions in total), 20-30 minutes per session	Parallel parenting course consisting of 4 sessions	Peterman (2008)
Promoting Alternative Thinking Strategies (PATHS) – Preschool Curriculum	To promote children's social-emotional skills. This curriculum targets four domains: prosocial friendship skills, emotional understanding and emotional expression skills, self-control and problem-solving skills, including interpersonal negotiation and conflict resolution.	The curriculum is divided into 33 lessons delivered by teachers during circle time. These lessons include modelling stories and discussions, and use puppet characters, photographs, and teacher role-play demonstrations. Each lesson includes extension activities (e.g., cooperative projects and games), providing children with opportunities to practice the target skills with teacher support.	Teacher	9 weeks	-	Arda (2012)
			Teacher	33 lessons, one per week over the course of one school year	Three 'take-home' packets provided to parents over the year. Each included a modelling videotape, parenting tips, learning activities and activities for home. Children also took home letter stickers and compliment pages to prompt their parents to ask them about their school day and provide positive support at home.	Bierman (2008)<br><br>Bierman (2014)

© 2018 Blewitt C et al. *JAMA Network Open.*

Intervention	Program Aim	Skills Targeted/Program Approach	Person Delivering Program	Intensity and Program Length	Parental Involvement	Relevant Studies
			Teacher	Once a week (30 lessons) plus extension activities for 9 months	-	Domitrovich (2007)
			Teacher	20 minutes, twice per week for 22 weeks	Frequent parent updates on curriculum content and suggestions for within the home.	Fishbein (2016)
			Teacher	Weekly, 15-30 minutes for 6 months	-	Hamre (2012)
			Teacher	1-2 lessons per week (44 in total) for 9 months	-	Hughes (2015)
			Teacher	1-2 times per week for the school year	-	Lonigan (2015)
			Teacher	Weekly lessons (30 in total) in circle time plus extension activities	-	Morris (2014)
			-	3 days per week for 90-120 minutes, over 16 weeks	-	Saltali (2010)
			Teacher	33 lessons (15-20 minute each) for 9 weeks	-	Seyhan (2017)

© 2018 Blewitt C et al. *JAMA Network Open.*

Intervention	Program Aim	Skills Targeted/Program Approach	Person Delivering Program	Intensity and Program Length	Parental Involvement	Relevant Studies
Promoting Social Competence in Classrooms (PSC)	To improve social competence.	The program teaches children problem-solving strategies and social and emotional skills. Stories involving puppets are presented to children to stage the demonstration of different skills and enable problem-solving.	Teacher and facilitator	2 workshops per month, 30 minutes per workshop. Program designed for 15 sessions, 9 sessions delivered	-	Moisan (2014)
Ready to Learn	Focused on the prerequisite learning skills cited most frequently in research literature as predictors of long-term school success: attending, listening comprehension and social skills.	Teachers use five strategies: modelling-coaching-curing, peer reporting, story-telling, story retelling and the encouragement council.	Teacher	Daily for 12 weeks	-	Brigman (2003)
			Teacher	2 hours per week, reinforced throughout the week for 12 weeks	-	Brigman (1999)
Red Light Purple Light	To improve behavioural self-regulation.	Focuses on circle time games. Participating children taken out of the playroom for each session. Each session began with a greeting song and ended with a goodbye classroom. Six activities (games) presented over the 16 sessions, that increased in complexity over time. Each game required attention and working memory and repeated at subsequent sessions.	Researcher	16 playgroups over 8 weeks. Each session approx. 30 mins. Each session included 5-8 children.	-	Tominey (2011)
Responsible Early Childhood Curriculum plus explicit social-	Explicit SEL component: To focus on social and emotional competencies related to success in school.	Whole-class circle time focusing on four units: understanding feelings, making friends, building self-esteem and increasing self-competence. For each unit, between five and seven books and a menu of activities were provided.	Teacher	Daily for 36 weeks	Parent newsletters sent home at the beginning of each 4- to 6-week unit describing the curriculum.	Landry (2014)

© 2018 Blewitt C et al. *JAMA Network Open.*

Intervention	Program Aim	Skills Targeted/Program Approach	Person Delivering Program	Intensity and Program Length	Parental Involvement	Relevant Studies
emotional classroom activities						
Second Step Preschool/ Kindergarten	A violence prevention curriculum intended to help children learn prosocial skills and reduce impulsive aggressive behaviour.	The Second Step curriculum is based within three units: empathy, management of emotions and resolution. Includes lesson cards accompanied by a manual. In addition, the kit includes posters, cards, puppets, a music CD, and take-home activities.	Teacher	25 lessons, twice per week for 25-50 minutes per lesson, over 3 months	Newsletter sent home	Amesty (2009)
			Researcher	25 lessons, twice per week for 30 minutes per lesson, over 4 months	-	Jack (2009)
			Teacher	15-25 minutes weekly for 7 months	-	Jakob (2005)
			Teacher	30 minutes per day across academic year	-	Starnes (2017)
			Researcher/nu rse	2 times per week, 30 minutes per session for 12 weeks	-	Stephenson (2009)
			Teacher	4 times weekly for 15 minutes, for approx. 22 weeks (delivery schedule adapted from publisher	Four to six parent-group sessions were provided each year	Upshur (2013)

© 2018 Blewitt C et al. *JAMA Network Open.*

Intervention	Program Aim	Skills Targeted/Program Approach	Person Delivering Program	Intensity and Program Length	Parental Involvement	Relevant Studies
				recommended weekly schedule)		
Second Step Early Learning (SSEL) Curriculum	Incorporates instruction and activities that address both social and emotional competence and executive functioning, including emotion knowledge and regulation, perspective taking and empathy, and social problem-solving skills, as well as attention, working memory, and inhibition.	Five major units: skills for learning, empathy, emotion management, friendship skills and problem solving, and transition to kindergarten. 28 weekly themes with different activities for each day of the week, theme-related songs and Brain Builder games intended to be played every day. Beyond the scripted daily activities, there are also suggested teaching strategies designed to reinforce skill growth.	Teacher	Daily, main daily activity 5-7 mins, plus strategies to integrate throughout the day. Pre and post measurements conducted in Sept – Nov and Mar – May over 2 years.	Parent handout for each weekly theme, called Home Link, that describes what the children are learning and ways parents can reinforce the themes at home.	Upshur (2017)
Self – Compassion and Mindfulness Training	To increase school-readiness skills.	The intervention included a variety of activities, demonstrations, and role-plays. Music, storybooks and deep breathing used before and after the main activity for each session. Topics included self-compassion, mindfulness, coping, compassion and managing feelings.	Principal Investigator	2 times per week, 15-30 minutes per session for 11 sessions, delivered to small group	-	Garrison (2017)
Self-Determination Program	Focus on three adaptive skill areas: direction following, sharing, and problem solving.	Stories focused on specific skills, taught through role-playing activities embedded in each story. Puppet games used as learning tools to reinforce individual skill. Each story formatted to introduce the major character, identify the problem, outline steps to solve, solve the problem. Teachers selected situations throughout each day when children could practice	Master's level student with support of teacher<br><br>1	Two 3-hour sessions per week for 12 weeks	Parent training sessions held during each of the major skill lessons (3 sessions, every 4 weeks). Storybooks sent home to enable parents to work on content with their child.	Serna (2000)

© 2018 Blewitt C et al. *JAMA Network Open.*

Intervention	Program Aim	Skills Targeted/Program Approach	Person Delivering Program	Intensity and Program Length	Parental Involvement	Relevant Studies
		their newly learned skills in the classroom environment.	Teacher	Two 3-hour sessions per week for 14 weeks	-	Serna (2003)
Social Emotional Intervention (includes components based on ICPS + PATHS)	Multifaceted program that targeted children's relationship with their teacher, emotional understanding, social problem solving with an emphasis on individualisation.	Activities derived from several sources. Activities on understanding emotions held two days per week. These activities reflect a downward extension of the PATHS curriculum, focusing on sympathy to the feelings of others and pro-social behaviour. I Can Problem Solve activities used for other 2 days during the week.	Teacher	4 days per week, 20 minutes per session, for 32 weeks	Newsletters sent home and parents' night out skits.	Denham (1996)
Social Emotional Learning Facilitator Kit (SELF Kit)	To develop social and emotional competencies in kindergarten and primary school children.	Program focused on 8 dysfunctional emotions: sadness/depression, separation anxiety, and fear of emotional injury /of being hurt, anger, guilt, shame, jealousy and envy. Each emotion is presented in a module that includes story-telling, a folktale, one or more therapeutic activities and games.	Teacher	1 module per week for 8 weeks	-	Opre (2013)
Social Emotional Prevention Program	To develop emotional competencies (emotion recognition and emotion regulation) and social competencies (compliance with rules, problem solving, and prosocial behaviours, such as turn-taking, toy sharing, and play cooperation).	Includes a series of modules. Begins with an introduction to classroom rules as the starting point for building more complex competencies such as emotion regulation, conflict resolution and regulatory abilities. The final module integrated children's previously acquired knowledge about emotions, emotion regulation, and prosocial behaviours in the context of children's peer play interactions. Methods include stories, puppet shows, role-playing and games.	Two teachers in each room	Daily for 4 months	The parent training included four group sessions combining information with coaching strategies for supporting social and emotional competence development and positive discipline.	Stefan (2013)
Social-Emotional	Constructed around themes related to awareness of emotions,	-	-	-	-	Deacon (2012)

© 2018 Blewitt C et al. *JAMA Network Open.*

Intervention	Program Aim	Skills Targeted/Program Approach	Person Delivering Program	Intensity and Program Length	Parental Involvement	Relevant Studies
Intervention	identification of emotions, and linking emotions with vocabulary.					
Stop and Think	To teach prosocial skills to children including survival skills, interpersonal skills, problem-solving skills, and conflict resolution.	Includes modelling, prompting, role-play, group activities and feedback. Using a process called "Stop & Think" children learn to verbalise and then to internalise five sequential steps: think about the situation, choose an appropriate plan of action, develop a specific plan to address the situation, implement the plan and self-reinforce appropriate behaviour.	Psychology graduate-level trainees	Two 30 minute sessions per week, for 10 weeks	-	Hall (2008)
			Teacher, with school psychology staff support for the first 3 lessons	Three 30 minute lessons with coach, then 15 minutes per week	-	King (2001)
Strong Start Pre-K	To promote social and emotional competence and reduce internalising problem behaviours.	Lessons cover specific objectives and goals that help to prevent emotional and mental health problems and develop a vocabulary to express feelings. Children's literature related to the relevant SEL topic is part of each lesson. A stuffed animal serves as a mascot to help contribute to scenarios.	Teacher	10 lessons in total, approx. 2 lessons per week at a convenient time	Bulletin sent home to parents at the end of each lesson outlining the content of the lesson and providing parents with strategies to reinforce social-emotional learning at home.	Gunter (2012)
Taking Part	-	Addresses skills essential to social development including listening to others, speaking kindly, using courtesy words, respecting others property, winning and losing with an emphasis on social communication, developing group play skills, and resolving conflicts.	Researcher	2 times per week for 45 minutes to 1 hour, for 15 weeks	After completing a unit, letters were sent home to inform parents about the social skills that were being taught to their children. If the child did not demonstrate this skill at home, the letter was sent back informing the teacher of the area of difficulty.	McKinney (1998)

© 2018 Blewitt C et al. *JAMA Network Open.*

Intervention	Program Aim	Skills Targeted/Program Approach	Person Delivering Program	Intensity and Program Length	Parental Involvement	Relevant Studies
The Incredible Years<br><br>Child Training Curriculum (Dinosaur School)	To promote children's social competence, emotional self-regulation and school behaviour.	Child Program:<br>Content broken into 7 units: learning school rules; how to be successful in school; emotional literacy, empathy, and perspective taking; interpersonal problem solving; anger management; social skills; and communication skills. Teachers use group circle time followed by small group skill practice. There are over 300 small group activities focusing on social emotional skills. Program includes life-size puppets, dinosaur homework activities, picture cue cards for non-readers, and games to stimulate group discussion, cooperation and skill-building.	Researcher	2 hours per week for 22 weeks	Included letters, homework and meeting at regular intervals.	Dereli (2009)
			Teacher	2 times per week, 35-40 minutes per session for 30 sessions (over approx. 15 weeks). Delivered to classroom and small group. Implemented in kinder and G1	Parents of a group of moderately high-risk children participated in The Incredible Years Parent discussion groups	Reid (2007)
			Teacher/ research staff member co-led.	At least 2 times per week, using 15–20-minute large group Circle time followed by 20 minutes of small group skill-practice activities. 30 lessons in curriculm	Weekly dinosaur homework to encourage parents' involvement.	Webster-Stratton (2008)<br><br>Teacher and child training
Creating a New Generation of Peacemakers	Violence prevention<br><br>Conflict avoidance, conflict resolution skills, respect for others, positive relationship skills	Each lesson begins with circle time. Lesson is presented using visual aids and children provide their own ideas and solutions. Lesson reinforced by individual or small group activities. Each lesson	2 trained facilitators	1hr per week for 5 weeks	Parents receive materials about the program before the first lesson and weekly summaries of each	Allen (2009)

© 2018 Blewitt C et al. *JAMA Network Open.*

Intervention	Program Aim	Skills Targeted/Program Approach	Person Delivering Program	Intensity and Program Length	Parental Involvement	Relevant Studies
(Peacemakers Program)		ends with the K.C (kind and caring) dog puppet interacting with the children in their group circle to reinforce the skills from the lesson.			lesson with suggestions for home activities.	
The Positive Action Program	To promote and strengthen positive behaviours, taking an asset-building approach to intervention and prevention.	Includes visuals and hands-on experiences. Based on six units: understanding of PA and self-concept; physical health and intellectual health; self-management and self-control; respect of others, consideration of others, and social bonding; honesty with self and others; and self-improvement.	Teacher	Daily for 10-15 minutes over 10 weeks	-	Schmitt (2014)
			Teacher	Daily 10-15 minutes (64 lessons) over 15 weeks	-	Schmitt (2017)
The Early Impact Program	To address the development of conduct problems in preschool-aged children.	Involved training teachers to implement strategies that could be universally applied. The curriculum focuses on communication, friendship formation, social problem-solving, self-control, and engaging in pro-social behaviours.	Teacher	10 weeks	Parents of children in the intervention group encouraged to attend parent-training sessions, facilitated over three 120-minute meetings	Larmar (2006)
The Safe Kindergarten	Provide children with knowledge and tools to create safer effective interactions with peers.	Effective dialogue: conveying thoughts and feelings, and listening attentively to others when they are expressing their thoughts and feeling.<br><br>20 units that include Imago topics and practice using the dialogue tool. Sessions follow a cumulative, developmental curriculum with a 4-part structure that includes an introduction, presentation of a theoretical issue and systematic practice of the intentional dialogue.	Teacher	20-30 mins per week for 20 weeks, delivered to small group (approx. 6 children)	A month after the program began, parents participated in a 2-hour workshop that introduced the Imago ideas and the program's aims and approach.	Aram (2008)

© 2018 Blewitt C et al. *JAMA Network Open.*

Intervention	Program Aim	Skills Targeted/Program Approach	Person Delivering Program	Intensity and Program Length	Parental Involvement	Relevant Studies
Tools of the Mind	Build broad foundational skills, regulating social and cognitive behaviors, attending and remembering on purpose, the use of symbolic representation, and early math and literacy skills.	Self-regulation. The program emphasises children's abilities to regulate their own social and cognitive behaviours, attend to and remember on purpose, use symbolic representation, and early math and literacy skills. Teachers support children to develop various psychological "tools" that help children to master their own psychological functions. The curriculum incorporates 40 Vygotsky-inspired activities designed to promote mature dramatic play, encourage the use of self-regulatory private speech, and teach the use of external aids to facilitate attention and memory.	Teacher	Embedded within the classroom during the year.	-	Barnett (2008)
Tools of the Mind - Play	To promote self-regulation through play.	Focuses on developing self-regulation through "pretend" or "make-believe" play where children are supported to use their imaginations to role-play, pretend they are different characters, play out different stories, and enact various scenarios that rely on and encourage creativity.	Teacher	Embedded within the classroom, includes daily 50-minute time block devoted to interactive pretend play, which is organised and scaffolded by teachers in very specific ways.	-	Morris (2014)
Values Education Programme	To learn positive social behaviours	-	Researcher	3 sessions per week (30 minutes each) for 11 weeks	Not directly - parents completed Family Participation Form that influenced content of program.	Dereli-Iman (2014)

© 2018 Blewitt C et al. *JAMA Network Open.*

Intervention	Program Aim	Skills Targeted/Program Approach	Person Delivering Program	Intensity and Program Length	Parental Involvement	Relevant Studies
You Can Do It! Early Childhood Education Program	Positive social, emotional, behavioural and achievement outcomes	Children provided with explicit instruction in five social-emotional competencies: Confidence, Persistence, Organisation, Getting Along, and Emotional Resilience, supported by explicit teaching of 12 ways of thinking ('Habits of the Mind'), I Can Do It, Accepting Myself, Taking Risks, Being Independent, Giving Effort, Working Tough, Setting Goals, Planning My Time, Being Tolerant of Others, Thinking First, Playing by the Rules, and Being Socially Responsible.	Teacher	Three 20 minute sessions per week for 2 weeks	-	Ashdown (2011)
Zippy's Friends	Mental health promotion program to teach children coping skills.	Built around six illustrated stories about a group of young children and a pet insect called Zippy. Sessions divided into six modules, each focusing on a theme (feelings, communication, making and breaking relationships, conflict resolution, dealing with change and loss and coping). Each session has specific goals and includes 2–3 participatory activities.	Teacher	Once per week for 24 weeks	-	Mishara (2006)
			Teacher	Weekly one hour sessions for 24 weeks	-	Rodker (2013)

© 2018 Blewitt C et al. *JAMA Network Open.*

eTable 3. Summary of Constructs within each Domain of Social-Emotional Development and Measures Used

Domain	Construct	Measures Used by Studies Included in this Review
Social Competencies	Pro-Social Skills<br><br>*Covers Social Knowledge, Social Problem Solving, Sharing, Social Cooperation, Social Interaction, Social Independence, Assertion, Responsibility. Measures of Social-Emotional Competence and School Readiness noted in this category*	ACER Well-Being Survey (Teacher Form— Early Years), Adaptive Social Behavior Inventory, Behavioral and Emotional Rating Scale, Berkeley Puppet Interview, Challenging Situations Task, Cooper-Farran Behavioral Rating Scales, Devereux Early Childhood Assessment Clinical Form (Parent and Teacher Report), Drexel Early Childhood Behavior Rating Scale, Early Screening Project, Hahnemann Behavior Rating Scale, Measure developed for study, Minnesota Preschool Affect Checklist (Adapted), Mize & Ladd Enactive Social Knowledge Interview, Observations, PED Cognition Test Battery, Platform for Child Development Evaluation, Playful Situations Task, Preschool and Kindergarten Behavior Scales, Preschool Behaviour Questionnaire, Preschool Competence Questionnaire, Preschool Interpersonal Problem-Solving Test, Preschool Social Behavior Questionnaire, Preschool Social Behavior Scale, Pro-Social Orientation Story-Completion Task, School Readiness and Conduct Problems: Coder Observation of Adaptation-Revised (COCA-R), Sharing Task, Social Competence and Behavior Evaluation, Social Competence Scale, Social Problem Solving Test, Social Problem Solving Test (modified), Social Skills Evaluation Scale, Social Skills Improvement System, Social Skills Questionnaire, Social Skills Rating System, Strengths and Difficulties Questionnaire, Student Rating Scale, The Head Start Competence Scale, The Preschool Promoting Alternative Thinking Strategies Evaluation Kit, The Playful Solutions Task, The Social Competence Screening for Pre-schoolers - Teacher Form, The Student Rating Scale, Wally's Problem Solving Test.
	Relationships and Peer Skill	Adult-Child Relationship Scale, Devereux Early Childhood Assessment Clinical Form (Parent and Teacher Report), North Carolina Family Assessment - Preschool Version, Minnesota Preschool Affect Checklist (Adapted), Multiple Option Observation System for Experimental Studies (MOOSES), Peer Relations Questionnaire, Preschool Competence Questionnaire, Semi-Structured Play Interview, Student-Teacher Relationship Scale.
	Communication	Role play of dialogue between friends, The Vineland Screener.
	Social Information Processing	Structured interview to map social information processing (Tur-Kaspa & Bryan 1994).
Emotional Competencies	Emotional Skills	Assessment of Children's Emotion Skills, Emotional Competence Screening for Pre-schoolers - Parent Form, Behavioural and Emotional Rating Scale, FEEK (Fragebogen zur Erfassung emotionaler Kompetenzen; Emotional Competencies Questionnaire), PED Cognition Test Battery, Schoolagers Coping Strategies Inventory, Second Step Interview, The Sullivan Teacher Rating Scale of Emotional Intelligence for Children, Wally's Feelings Test.

© 2018 Blewitt C et al. *JAMA Network Open.*

	Emotion Knowledge/Understanding	Affective Knowledge Test, Affective Knowledge Test (modified), Assessment of Children's Emotion Skills, Emotion Knowledge Inventory, Emotion Matching Scale/Emotion Matching Task, Emotion Recognition Measure, Emotion Recognition Questionnaire, Emotional Understanding Measure Adapted from Bullock and Russell Test of Emotion Comprehension, Facial Emotions Identification and Emotions Situations Tasks, NEPSY - Second Edition, Test of Emotion Comprehension, The Sullivan Emotional Intelligence Scale for Children, Wally Understanding Feelings Test.
	Empathy	Teacher Behavior Form, The Attachment Story Completion Task, The Distress Task, The Sullivan Brief Empathy Scale for Children.
	Emotional Vocabulary	Emotion Labelling Measure, Kusche Emotional Inventory.
	Emotional Regulation	Emotion Regulation Checklist, Head Start Competence Scale (Teacher Version), Preschool Behavioral and Emotional Rating Scale, Social Competence Scale.
	Emotional Expression/Affect/Affective Perspective Taking	Assessment of Children's Emotion Skills, Assessment of Children's Emotions Scales, Denham Puppet Interview, Emotion Expression Rating Scale, Minnesota Preschool Affect Checklist (Adapted), NEPSY-II Social Perception Subtests, Observations.
Behavioural Regulation	Learning Engagement	Measure designed for study.
	Positive Behaviour/Behavioural Response	ADD-H Comprehensive Teacher's Rating Scale, Challenging Situations Test, Comprehensive Teacher's Rating Scale, Cooper-Farran Behavioral Rating Scales, Early Screening Project, Independent observations of attending behaviour or positive behaviour, Psycho-Social Behaviour Scale for Pre-school Children.
	Self-Control	Devereux Early Childhood Assessment Clinical Form, Devereux Early Childhood Assessment Clinical Form - Teacher Report, Social Skills Questionnaire, Social Skills Rating System, The Head-Toes-Knees-Shoulders Task.
Problem Behaviours and Emotions	Aggression	Aggressive Behavior Scale, Berkeley Puppet Interview, Caregiver-Teacher Report Form, Challenging Situations Task, Child Behavior Checklist - Teacher Report Form, Direct assessment that measured social problem solving (Alber et al. 1995), Devereux Early Childhood Assessment Clinical Form, Devereux Early Childhood Assessment Clinical Form – Teacher Report, Drexel Early Childhood Behavior Rating Scale, Early Screening Project, Early Years Behaviour Checklist, Enactive Social Knowledge Interview (Mize & Ladd), Hahnemann Behavior Rating Scale, Observations, Preschool and Kindergarten Behavior Scale, Preschool Behaviour Scale, Preschool Interpersonal Problem-Solving Test (force), Preschool Social Behavior Scale, Revised Aggression Scale, Social Competence and Behavior Evaluation, Social problem

© 2018 Blewitt C et al. *JAMA Network Open.*

		solving direct assessment, Teacher Observation of Child Adaptation-Revised, The Preschool Promoting Alternative Thinking Strategies Evaluation Kit.
	Introversion/ Withdrawal/Anxiety	Caregiver-Teacher Report Form, Challenging Situations Task, Child Behavior Checklist - Teacher Report Form, Devereux Early Childhood Assessment Clinical Form, Devereux Early Childhood Assessment Clinical Form – Teacher Report, Drexel Early Childhood Behavior Rating Scale, Hahnemann Behavior Rating Scale, Multiple Option Observation System for Experimental Studies (MOOSES), Observations, Preschool and Kindergarten Behavior Scales, Preschool Bullying Subscales Measure, Social Competence and Behavior Evaluation, Strengths and Difficulties Questionnaire, The Preschool Anxiety Scale.
	Problem Behaviours	Adaptation of the Minnesota Preschool Affect Checklist, Behavior Problems Index, Caregiver-Teacher Report Form, Devereux Early Childhood Assessment Clinical Form, Devereux Early Childhood Assessment Clinical Form - Teacher Report, Dyadic Parent-Child Interactive Coding System, Early Screening Project, Multiple Option Observation System for Experimental Studies (MOOSES), North Carolina Family Assessment - preschool version, Observations, Preschool and Kindergarten Behavior Scales, Preschool Social Behaviour Scale, Preschool Social Behavior Questionnaire, Strengths and Difficulties Questionnaire (Parent and Teacher version), Social Competence and Behavior Evaluation - Preschool Edition, Social Problem Solving Test, Social Skills Improvement System, Social Skills Rating System, Sutter-Eyberg Student Behavior Inventory – Revised, The IOWA Conners, The Teacher-Child Rating Scale.
	Conflict	Adult-Child Relationship Scale, Preschool Behavior Scale - Teacher Rating Strengths and Difficulties Questionnaire, Student-Teacher Relationship Scale.
	Externalising	Caregiver-Teacher Report Form, Child Behavior Checklist -Teacher Report Form, Preschool Behavior Questionnaire, Preschool Social Behavior Questionnaire, Social Competence and Behaviour Evaluation - Preschool Edition, Social Skills Rating System, Teacher Observations, The Preschool and Kindergarten Behavior Scales.
	Internalising	Caregiver-Teacher Report Form, Child Behavior Checklist -Teacher Report Form, Preschool and Kindergarten Behavior Scales, Social Competence and Behaviour Evaluation - Preschool Edition, Social Skills Rating System, Teacher Observation of Child Adaptation-Revised, Teacher Observations, The Preschool and Kindergarten Behavior Scales
	Emotional Reactivity/Emotional Control Problems	Caregiver-Teacher Report Form, Child Behavior Checklist - Teacher Report Form, Devereux Early Childhood Assessment Clinical Form, Devereux Early Childhood Assessment Clinical Form – Teacher Report, Emotion Regulation Checklist, Social Skills Rating System, Strengths and Difficulties

© 2018 Blewitt C et al. *JAMA Network Open.*

		Questionnaire (Parent and Teacher version), The Emotion Expression Ratings Scale, Teacher Rating of Negative Emotions.
	Anxiety/Depression	Berkeley Puppet Interview, Caregiver-Teacher Report Form, Child Behavior Checklist - Teacher Report Form, Platform for Child Development Evaluation, Preschool and Kindergarten Behavior Scale, Preschool Anxiety Scale.
	Behaviour Inhibition	Behavioral Inhibition Questionnaire, Behavior Inhibition Scale.
	Attention Problems	ADHD Rating Scale, Caregiver-Teacher Report Form, Child Behavior Checklist - Teacher Report Form, Devereux Early Childhood Assessment Clinical Form, Devereux Early Childhood Assessment Clinical Form – Teacher Report, Multiple Option Observation System for Experimental Studies (MOOSES), Preschool and Kindergarten Behavior Scale, Preschool Social Behaviour Scale, Social Competence Scale, Social Skills Rating System, Strengths and Difficulties Questionnaire, Teacher Observations, The Abbreviated Conners Rating Scale, The IOWA Conners.
Early Learning Outcomes	Reading/Literacy	Admission Assessment for Beginning Learners, Get Ready to Read, Teacher report, Test of Preschool Early Literacy, The Preschool Comprehensive Test of Phonological and Print Processing, Woodcock-Johnson Letter-Word Identification Test.
	Language	Admission Assessment for Beginning Learners, Expressive One-Word Picture Vocabulary Test, Peabody Picture Vocabulary Test-III, Preschool Language Scale, Test of Language Development, The Primo Vocabolario del Bambino, Woodcock-Johnson Picture Vocabulary Test.
	Listening	Metropolitan Readiness Test, Stanford Early School Achievement Test.
	Comprehension/Understanding of Vocabulary	Metacognitive Verb Comprehension, Test di Valutazione del Linguaggio (Test of Language Evaluation).
	Math Skill	Admission Assessment for Beginning Learners, Child Math Assessment, Woodcock-Johnson Applied Problems.
	Academic Competence	Academic Rating Scale, Berkeley Puppet Interview, North Carolina Family Assessment - Preschool Version, Preschool Competence Questionnaire, Social Skills Rating System - Teacher Questionnaire.

© 2018 Blewitt C et al. *JAMA Network Open.*
